# Addressing the estimation of standard errors in fixed effects meta‐analysis

**DOI:** 10.1002/sim.7625

**Published:** 2018-03-25

**Authors:** Clara Domínguez Islas, Kenneth M. Rice

**Affiliations:** ^1^ Fred Hutchinson Cancer Research Center Seattle WA USA; ^2^ MRC Biostatistics Unit School of Clinical Medicine, University of Cambridge Cambridge UK; ^3^ Department of Biostatistics University of Washington Seattle WA USA

**Keywords:** fixed effects, heterogeneity, meta‐analysis, random effects

## Abstract

Standard methods for fixed effects meta‐analysis assume that standard errors for study‐specific estimates are known, not estimated. While the impact of this simplifying assumption has been shown in a few special cases, its general impact is not well understood, nor are general‐purpose tools available for inference under more realistic assumptions. In this paper, we aim to elucidate the impact of using estimated standard errors in fixed effects meta‐analysis, showing why it does not go away in large samples and quantifying how badly miscalibrated standard inference will be if it is ignored. We also show the important role of a particular measure of heterogeneity in this miscalibration. These developments lead to confidence intervals for fixed effects meta‐analysis with improved performance for both location and scale parameters.

## 1 INTRODUCTION

Meta‐analysis, “the use of statistical methods to summarize the results of independent studies,”^1^, ^2^ is a pivotal component of systematic reviews^2^ that have been extensively used to synthesize the increasing amount of evidence produced in health care research.^3^ In broad terms, the primary aim of most meta‐analyses is to make some form of inference on the size of effects across several similar studies. A typical goal is to summarize all the studies and make inference on the magnitude and direction of some form of average effect. Measures of spread, ie, how the study effects vary across different studies, are also often considered.

A recent review paper^4^ notes that the well‐known inverse‐variance fixed effects estimate, which can be easily motivated as an estimate of a “common effect,” can also be interpreted as a particular average of study‐specific effects, without any requirement that study effects be homogeneous. However, using this alternative interpretation is only straightforward when the standard errors are known with negligible error, a simplifying assumption that is rarely entirely plausible in practice. The impact of this assumption has been studied in some special case (ie, when assuming homogeneity),^5^, ^6^ but a general understanding of how fixed effects meta‐analysis is affected is missing from the literature. In the present work, we therefore provide several tools to do statistical inference for fixed effects meta‐analysis when this assumption cannot be made.

The paper is structured as follows: in Section 2, we review the different statistical models that can be used for meta‐analysis, their parameters of interest, and popular estimation methods. In Section 3, we show how the precision weighted average effect arises naturally when considering optimal summary measures and also propose a measure of heterogeneity around this parameter. In Section 4, we present intuitive and formal arguments for why the impact of standard error estimation does not go away with larger sample sizes and how this impact depends on the underlying heterogeneity. We present simulation results comparing several confidence intervals for the precision weighted average that allow for estimation of standard errors, and for a related measure of heterogeneity. Finally, in Section 5, we give an applied example to illustrate and compare the different approaches to meta‐analysis and conclude with a discussion in Section 6.

## 2 REVIEW OF APPROACHES TO META‐ANALYSIS

In this section, we describe 3 different approaches to meta‐analysis, in which different assumptions are made about the underlying true effect size parameters in the studies. Table 1 provides a summary of these approaches, and we subsequently present further details on the precision weighted average (Section 2.1), testing and quantifying heterogeneity (Section 2.2), and random effects analysis (Section 2.3).

**Table 1:** Statistical assumptions from 3 different approaches to meta‐analysis of k studies, their target parameters for location summary and estimators

Common	${\hat{\beta }}_{i}∼N(\beta_{i},\sigma_{i}^{2})$, with $\sigma_{i}^{2}$ known, for i=1,2,…,k		
assumption
Approach‐specific	**Common Effect**	**Fixed Effects**	**Random Effects**
assumption	$\beta_{i}=\beta_{0}\forall i,\beta_{0}\in {I\;R}^{k}$	$\beta ={\left(\beta_{i},\text{⋯},\beta_{k}\right)}^{T}\in {I\;R}^{k}$	β_1_,…,β_k_ iid f(μ,τ ^2^)
Inference target	β_0_	$\beta_{F}=\frac{\sum_{i=1}^{k}\frac{1}{\sigma_{i}^{2}}\beta_{i}}{\sum_{i=1}^{k}\frac{1}{\sigma_{i}^{2}}}$	μ
Estimator	${\hat{\beta }}_{0}=\frac{\sum_{i=1}^{k}\frac{1}{\sigma_{i}^{2}}{\hat{\beta }}_{i}}{\sum_{i=1}^{k}\frac{1}{\sigma_{i}^{2}}}$	${\hat{\beta }}_{F}=\frac{\sum_{i=1}^{k}\frac{1}{\sigma_{i}^{2}}{\hat{\beta }}_{i}}{\sum_{i=1}^{k}\frac{1}{\sigma_{i}^{2}}}$	$\hat{\mu }=\frac{\sum_{i=1}^{k}\frac{1}{\sigma_{i}^{2}+{\hat{\tau }}^{2}}{\hat{\beta }}_{i}}{\sum_{i=1}^{k}\frac{1}{\sigma_{i}^{2}+{\hat{\tau }}^{2}}}$
Standard error	$\hat{\text{SE}}({\hat{\beta }}_{0})=\sqrt{\frac{1}{\sum_{i=1}^{k}\frac{1}{\sigma_{i}^{2}}}}$	$\hat{\text{SE}}({\hat{\beta }}_{F})=\sqrt{\frac{1}{\sum_{i=1}^{k}\frac{1}{\sigma_{i}^{2}}}}$	$\hat{\text{SE}}(\hat{\mu })=\sqrt{\frac{1}{\sum_{i=1}^{k}\frac{1}{\sigma_{i}^{2}+{\hat{\tau }}^{2}}}}$
Heterogeneity	Not present,	Hypothesis test based on	${\hat{\tau }}^{2}=\max\left\{0,\frac{Q-(k-1)}{\sum \sigma_{i}^{-2}-\sum \sigma_{i}^{-4}/\sum \sigma_{i}^{-2}}\right\}$
	by assumption	$Q=\sum_{i=1}^{k}\frac{1}{\sigma^{2}}{\left({\hat{\beta }}_{i}-{\hat{\beta }}_{F}\right)}^{2}$	
Consistency	Not evaluated	$I^{2}=\frac{Q-(k-1)}{Q}$	$I^{2}=\frac{Q-(k-1)}{Q}$

The first approach is the fixed effect (singular) meta‐analysis, also called the common effect meta‐analysis.^4^ This approach is based on the assumption of a single, common effect underlying all studies.^6^ Under this simplifying assumption that all study effects are identical, the average effect is equivalent to the common effect size estimated in each study. Although commonly used, this method has often been judged inadequate in practice, as effects from different studies are expected to differ given the variability in study design, population, interventions, etc.^7^, ^8^, ^9^

A second approach is the fixed effects (plural) meta‐analysis, based on the assumption that the effects underlying the studies at hand are unknown, but fixed, and not necessarily identical.^10^, ^11^ Using the fixed effects approach, it is common to estimate the inverse‐variance weighted average of the studies' effect sizes,^4^ but estimation of other weighted averages is also possible.^11^, ^12^ As recently discussed by Rice et al,^4^ the inverse‐variance weighted average estimates a reasonable and interpretable parameter, even when the effect sizes are assumed to be different, but it may be a somewhat incomplete summary of the effect sizes if they are too heterogeneous.^13^

The third approach is the random effects meta‐analysis, where the effect‐size parameters are considered to be a random sample from a population, ie, they follow a probability distribution.^14^ By using random effects as a sampling model, this analysis allows the estimation of the average effect size in the population of effect sizes one might ever have observed.^14^ (Details are given in Section 2.3.) This method not only takes into account the heterogeneity between studies but also provides a natural way of quantifying it,^15^ making it a more attractive choice over the common and fixed effects approaches.^7^, ^8^ On the other hand, as pointed out by Higgins et al,^15^ this approach is based on a construct of an hypothetical population of studies or study effects, so the interpretation of the analysis is potentially unclear and confusing. The relevance of random effects analyses that focus on the mean of a population of study effects has been questioned.^16^ An alternative derivation for the random effects approach motivates the distribution of effect sizes not as a sampling distribution, but arising from a priori exchangeability in a Bayesian analysis—or an approximately Bayesian analysis, as noted in Higgins et al.^15^

In each of the 3 approaches described, appeals to some form of frequentist optimality can be made. In the common effect approach, when the study‐specific standard errors are known precisely, the optimality is straightforward; without any distributional assumptions, the inverse‐variance weighted estimator provides the best linear unbiased estimator of the common effect, or the unique minimum variance unbiased estimator under the further assumption of normality of effects estimates.^6^ When the study‐specific standard errors must be estimated, a normal approximation based on the asymptotic distribution of the estimator is commonly used^17^, chapter 6; in the common situation where all the studies are large, the standard errors are known with great accuracy, and any nonasymptotic inefficiency is extremely minor.

For the fixed effects approach, it has been shown by Lin and Zeng^18^ that the analysis provides, in many situations, a statistically efficient estimate of the parameter that would be estimated, were it possible to pool the data across studies and to perform a single regression analysis that adjusts for study. However, this pooling is inherently somewhat hypothetical; were it possible to do it, there would often be little motivation for use of meta‐analysis, and so it may not always be obvious that this parameter is of direct interest.

The random effects approach has perhaps the least direct connection to optimality, while likelihood‐based and fully Bayesian methods in general have guarantees of good large‐sample properties, under correct model assumptions,^19^, ^20^ in finite samples or when the model is misspecified there are no such guarantees. Indeed, the finite‐sample sensitivity of Bayesian random effects meta‐analysis to choice of priors is well documented^21^, ^22^, ^23^, ^24^ and is a cause for concern in practice.^25^, chapter 5

### 2.1 The precision weighted average

Let β_1_,β_2_,…β_k_ be the true effect sizes from k different studies and let ${\hat{\beta }}_{i}$ be the estimate of the true effect β_i_, with corresponding standard error σ_i_, which we assume known for now. The precision weighted average or inverse‐variance weighted average of the true effect sizes is

(1) $$\beta_{F}=\frac{\sum_{i=1}^{k}\frac{1}{\sigma_{i}^{2}}\beta_{i}}{\sum_{i=1}^{k}\frac{1}{\sigma_{i}^{2}}}.$$

This parameter is a quantity of interest in either common effect or fixed effects meta‐analysis; under the common effect model, β_F_ reduces to the common effect β_0_ seen in Table 1; under the fixed effects model, β_F_ is a weighted average of the effect‐sizes β_i_, where the weight is proportional to the precision with which each effect size can be estimated, giving more weight to those that can be estimated more precisely

If the study‐specific standard errors σ_i_ are assumed to be known, a natural estimator of β_F_ is given by

(2) $${\hat{\beta }}_{F}=\frac{\sum_{i=1}^{k}\frac{1}{\sigma_{i}^{2}}{\hat{\beta }}_{i}}{\sum_{i=1}^{k}\frac{1}{\sigma_{i}^{2}}}\;\text{, with}\;\text{SE}({\hat{\beta }}_{F})=\sqrt{\frac{1}{\sum_{i=1}^{k}\frac{1}{\sigma_{i}^{2}}}}.$$

Optimality of β_F_ under a common effect approach has already been mentioned. In fixed effects meta‐analysis with known standard errors σ_i_, ${\hat{\beta }}_{F}$ directly inherits any efficiency properties from the ${\hat{\beta }}_{i}$'s, as would any linear combination of the effect‐size estimates. This means that ${\hat{\beta }}_{F}$ is an unbiased, efficient, and/or normally distributed estimator of β_F_, if within each study, the estimator ${\hat{\beta }}_{i}$ can be assumed to be an unbiased, efficient, and/or normally distributed estimator of β_i_.

Confidence intervals for β_F_ are usually derived from a normal approximation, appealing to the large sample properties of the study estimators. Transformations of the outcome measure have also been recommended, such as normalizations, log transformations, bias corrections,^17^ and/or variance stabilizing transformations.^26^, ^27^ The small sample properties of the normal approximation and sensitivity to the assumption of known variances have been studied through simulation studies,^17^, ^28^, ^29^ and some corrections and tests based on more robust test statistics have been proposed.^5^, ^29^

### 2.2 Testing homogeneity and quantifying heterogeneity

In both fixed effects and common effect work, it is common to test homogeneity of the study effects, that is, to test the null hypothesis H_0_:β_1_=β_2_=…=β_k_, against the general alternative of heterogeneity, where some β_i_ are not equal. In common‐effect meta‐analysis, this test assesses a key modeling assumption, while in the fixed effects analysis, the test simply gives a statistical measure of how much heterogeneity is present.

When assessing homogeneity, a commonly used test statistic is

$$Q=\sum_{i=1}^{k}\frac{1}{\sigma_{i}^{2}}{\left({\hat{\beta }}_{i}-{\hat{\beta }}_{F}\right)}^{2}.$$

Under normality of the effect estimates ( ${\hat{\beta }}_{i}∼N(\beta_{i},\sigma_{i}^{2})$), Q is distributed noncentral chi‐squared with k−1 degrees of freedom and noncentrality parameter

$$\lambda =\sum_{i=1}^{k}\frac{1}{\sigma_{i}^{2}}{\left(\beta_{i}-\beta_{F}\right)}^{2},$$

with β_F_ as in (1). Q is independent of the ${\hat{\beta }}_{F}$ statistic,^4^ which may simplify its interpretation.

Under the null hypothesis that all the effects are identical, the Q statistic is distributed central chi‐squared with k−1 degrees of freedom, thus providing a reference distribution to perform a test of homogeneity of effects. However, it also has been found that this test of homogeneity has low power when there are few studies^30^ and is not adequate to summarize the extent of the heterogeneity present.^31^

Other statistics have been proposed to not only test but also evaluate the impact of the observed heterogeneity, and thus provide a better measure of the consistency between trials.^31^ Although these measures have been motivated and derived from a random effects framework, they still have valid interpretation under a fixed effects framework.^4^ The most‐frequently used of these quantities is I ^2^, which can be calculated as

$$I^{2}=\frac{Q-(k-1)}{Q},$$

and is interpreted as “the percentage of total variation across studies that is due to heterogeneity rather than chance.”^32^

### 2.3 Inference in random effects meta‐analysis

Random effects meta‐analysis is based on the assumption that the true study effects β_1_,β_2_,...β_k_ are an independent and identically distributed sample from some distribution. The inference is then focused on the parameters of this distribution, typically its mean (μ) and variance (τ ^2^).

With no further assumptions on the distribution of the random effects, an inverse‐variance weighted average estimate of μ can be obtained,^14^, ^15^ along with an estimate of its standard error:

(3) $$\hat{\mu }=\frac{\sum_{i=1}^{k}\frac{1}{\sigma_{i}^{2}+{\hat{\tau }}^{2}}{\hat{\beta }}_{i}}{\sum_{i=1}^{k}\frac{1}{\sigma_{i}^{2}+{\hat{\tau }}^{2}}}\;\text{, with}\;\hat{\text{SE}}(\hat{\mu })=\sqrt{\frac{1}{\sum_{i=1}^{k}\frac{1}{\sigma_{i}^{2}+{\hat{\tau }}^{2}}}}.$$

The weights here involve both the within‐study variance $\sigma_{i}^{2}$ and the heterogeneity (or between studies) variance τ ^2^, for which a moment‐based estimator is

$${\hat{\tau }}^{2}=\max\left\{0,\frac{Q-(k-1)}{\sum \sigma_{i}^{-2}-\sum \sigma_{i}^{-4}/\sum \sigma_{i}^{-2}}\right\}.$$

As given here, $\hat{\mu }$ and $\hat{\tau }$ are known as the DerSimonian‐Laird estimator for random effects meta‐analysis.^14^ Other similar moment‐based estimator have been proposed.^33^, ^34^

Under the further assumption that the study effects follow a normal distribution, maximum likelihood^35^, ^36^ and restricted maximum likelihood^37^, ^38^ methods can be used to obtain estimates of τ ^2^ and μ. Although these methods are iterative and do not provide closed form estimates, it should be noticed that both the maximum likelihood and REML estimators of μ take the same form as in (3). A simpler, noniterative method for estimating τ ^2^ has recently been proposed^39^ and is also based on the assumption of a normal distribution of the study effects. The performance of the different estimation methods has been evaluated and compared, in terms of bias and efficiency,^34^ as well as coverage probability.^40^

## 3 UNDERSTANDING HOW ESTIMATION OF STANDARD ERRORS AFFECTS FIXED EFFECTS META‐ANALYSIS

In Equation 1 of Section 2.1, we saw how the underlying parameter estimated by fixed effects meta‐analysis is typically defined, in terms of standard errors. When the standard errors are not known but only estimated, this leaves the target of this analysis without a full definition. In Section 3.1, we provide a more concrete motivation, showing how the parameter estimated is optimal for inference, in a certain sense. The impact of estimated standard errors on this inference is explored in Section 3.2, and we see how this motivates the study of a particular scale parameter, describing heterogeneity, in Section 3.3.

### 3.1 A location parameter for optimal estimation

Ideally, the parameters to which inference is targeted should be determined entirely by scientific criteria, ie, by research goals. But in practice these goals may not be known precisely enough to determine a single parameter for inference. In this situation, it makes sense to use statistical criteria to choose from among parameters that meet general research goals. In meta‐analysis, where the general goal is to summarize study effects β_i_ by some form of average, we choose to pick the the affine combination (ie, the weighted average) of the β_i_ that can be most precisely estimated. This can also be stated as selecting the parameter for which the data provides the most information.

The main result here follows from a more general lemma, proved in Appendix A:

Lemma 1 Let $\left\{v^{T}\beta :v\in R^{k},v^{T}1_{k}=1\right\}$ be the set of all possible affine combinations of the vector of effect‐size parameters **β**=(β_1_,β_2_,…,β_k_)^T^ and let $\hat{\beta }$ be the vector of estimates ${\left({\hat{\beta }}_{1},{\hat{\beta }}_{2},\text{⋯},{\hat{\beta }}_{k}\right)}^{T}$ with covariance matrix **Σ**. Then the affine combination of the parameter vector (**w** ^T^ **β**) for which the corresponding estimator ( $w^{T}\hat{\beta }$) has the minimum variance is given by

$$w=\underset{v:v^{T}1_{k}=1}{\text{argmin}\;}\left[v^{T}\Sigma v\right]=\frac{\Sigma^{-1}1_{k}}{1_{k}^{T}\Sigma^{-1}1_{k}}$$

with $\mathrm{Var}(w^{T}\hat{\beta })={\left(1_{k}^{T}\Sigma^{-1}1_{k}\right)}^{-1}$.

In fixed effects meta‐analysis, where the studies are independent, the covariance matrix of $\hat{\beta }$ reduces to a diagonal matrix: **Σ**=diag{ $\sigma_{i}^{2}$}. From Lemma 1 and assuming that $\sigma_{i}^{2}$ is known exactly from each study, then the best affine combination of the effect‐size parameters is

(4) $$\left(\frac{1_{k}^{T}\text{diag}\{\sigma_{i}^{-2}\}}{1_{k}^{T}\text{diag}\{\sigma_{i}^{-2}\}1_{k}}\right)\beta =\frac{\sum_{i=1}^{k}\frac{1}{\sigma_{i}^{2}}\beta_{i}}{\sum_{i=1}^{k}\frac{1}{\sigma_{i}^{2}}}=\beta_{F},$$

the precision weighted average of the effect‐size parameters.

In the situation where the $\sigma_{i}^{2}$ are assumed known, the corresponding estimate ${\hat{\beta }}_{F}$ can be easily constructed, and used as described in Section 2.1.

But the same optimality of β_F_ holds even when the σ_i_ are not known. To show this formally, we express $\sigma_{i}^{2}$ as

$$\sigma_{i}^{2}={\left(n_{i}\phi_{i}\right)}^{-1}=N^{-1}{\left(\eta_{i}\phi_{i}\right)}^{-1},$$

where n_i_ and ϕ_i_ are the sample size and the Fisher information from each subject on β_i_, respectively, in the ith study, N is the total sample size across all studies, and η_i_=n_i_/N is the proportion of the total sample drawn from study i. Then formally, under the asymptotic regime where η_i_ are fixed when we consider larger N(ie, the same assumptions as in the earlier work of Lin and Zeng,^18^ and indeed most asymptotic work), then the limiting value of the covariance matrix is **Σ**=N ^−1^diag{(η_i_ ϕ_i_)^−1^}, and canceling terms in N, we find

$$\beta_{F}=\frac{\sum_{i=1}^{k}n_{i}\phi_{i}\beta_{i}}{\sum_{i=1}^{k}n_{i}\phi_{i}}=\frac{\sum_{i=1}^{k}\eta_{i}\phi_{i}\beta_{i}}{\sum_{i=1}^{k}\eta_{i}\phi_{i}}.$$

This shows that, without further assumptions, in large samples, β_F_ is the weighted average of the β_i_ parameters that can be most precisely estimated. When the true standard errors σ_i_ are not known but instead estimated by s_i_, β_F_ can be consistently estimated by a “plug‐in” version of ${\hat{\beta }}_{F}$ from Equation 2. We denote this estimate as

$${\hat{\hat{\beta }}}_{F}=\frac{\sum_{i}^{k}\frac{1}{s_{i}^{2}}{\hat{\beta }}_{i}}{\sum_{i}^{k}\frac{1}{s_{i}^{2}}}.$$

### 3.2 Impact of estimated standard errors on ${\hat{\hat{\beta }}}_{F}$ with estimated standard errors

When using the precision weighted average from Equation 2, it is common to assume that the sample size in each study is large enough for the variance of the effect estimate ( $\sigma_{i}^{2}$) to be approximated with negligible error by its estimate ( $s_{i}^{2}$),^41^ basing tests statistics and confidence intervals on the following plug‐in estimator:

(5) $${\hat{\hat{\beta }}}_{F}=\frac{\sum_{i}^{k}\frac{1}{s_{i}^{2}}{\hat{\beta }}_{i}}{\sum_{i}^{k}\frac{1}{s_{i}^{2}}}\;\text{, with}\;\hat{\text{SE}}({\hat{\hat{\beta }}}_{F})=\sqrt{\frac{1}{\sum_{i=1}^{k}\frac{1}{s_{i}^{2}}}}.$$

The properties of Equation 5 in small sample size settings have been studied via simulation, with inflated type I error rates observed for the test of the null hypothesis *H*_0_:*β*_*F*_=0, due to underestimation of the standard error of ${\hat{\hat{\beta }}}_{F}$.^17^, ^28^, ^29^ Corrected and alternative test statistics have been proposed,^5^, ^6^, ^29^ but all of them are based on the assumption of a common effect.

However, in our experience, many investigators expect that the effect of plugging‐in *s*_*i*_ for *σ*_*i*_ should, in large samples, be negligible for inference on *β*_*F*_, regardless of the underlying *β*_*i*_—and so the simulation results can be ignored when studies have large sample sizes. This intuition appears to be based on experience with other small‐sample corrections that change standard error estimates by factors of *n*/(*n*−1) or *n*/(*n*−*p*), which can be ignored with large *n*. However, this intuition does not apply to ${\hat{\hat{\beta }}}_{F}$; not only does the effect of plugging‐in *s*_*i*_ remain at any sample size, its impact depends importantly on the heterogeneity between the various *β*_*i*_.

To better understand how the potential heterogeneity affects the estimation of $\mathrm{Var}[{\hat{\hat{\beta }}}_{F}]$, we decompose the variance of ${\hat{\hat{\beta }}}_{F}$ as

(6) $$\begin{array}{ccc} \mathrm{Var}[{\hat{\hat{\beta }}}_{F}] & =E[\mathrm{Var}({\hat{\hat{\beta }}}_{F}|s_{1}^{2},\text{⋯},s_{k}^{2})]+\mathrm{Var}[E({\hat{\hat{\beta }}}_{F}|s_{1}^{2},\text{⋯},s_{k}^{2})] & \\ & =E\left[\left(\sum_{i}^{k}\frac{\sigma_{i}^{2}}{s_{i}^{4}}\right)/{\left(\sum_{i}^{k}\frac{1}{s_{i}^{2}}\right)}^{2}\right]+\mathrm{Var}\left[\left(\sum_{i}^{k}\frac{1}{s_{i}^{2}}\beta_{i}\right)/\left(\sum_{i}^{k}\frac{1}{s_{i}^{2}}\right)\right], \end{array}$$

where the second line follows from assumptions that each ${\hat{\beta }}_{i}$ is unbiased, is independent of its corresponding standard error estimate *s*_*i*_, and has variance $\sigma_{i}^{2}$.

**Figure 1:**
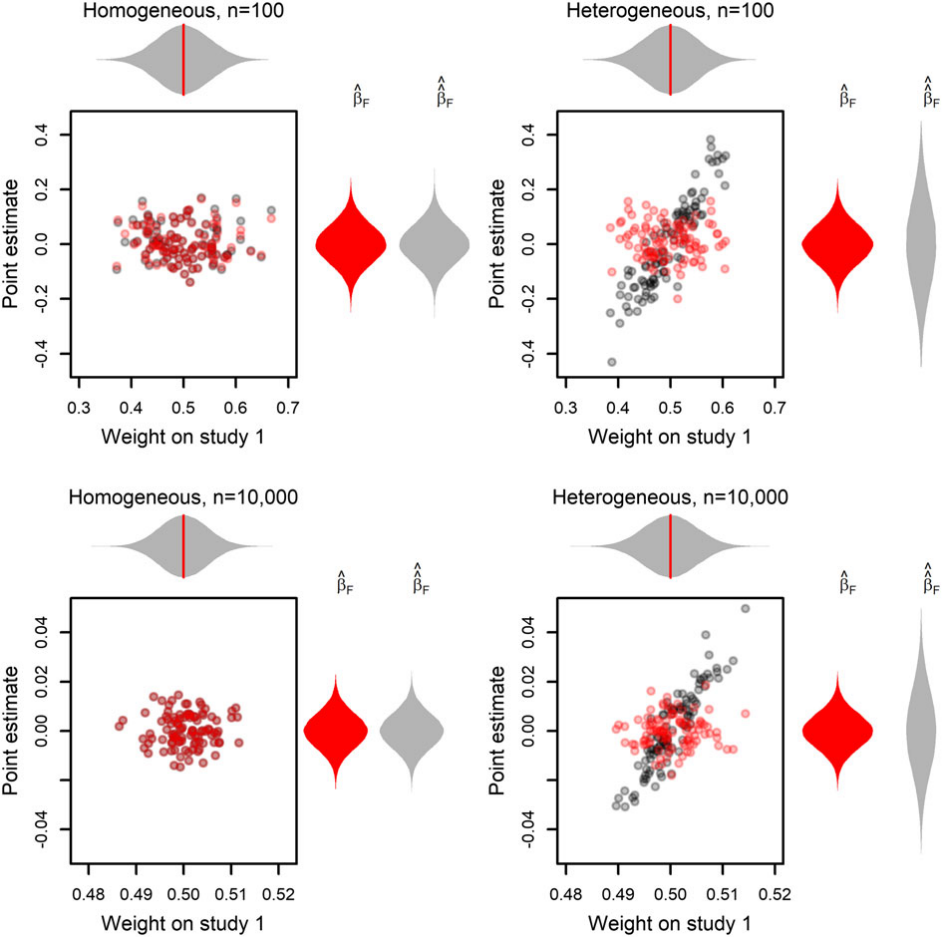
Comparison of the distributions of ${\hat{\beta }}_{F}$ and ${\hat{\hat{\beta }}}_{F}$ in a simple meta‐analyses of 2 homogeneous studies with effect sizes β_1_=β_2_=0 (left column) and 2 heterogeneous studies with effect sizes β_1_=1.5 and β_2_=−1.5 (right column). We consider medium size studies with N=100 (top row) and very large studies with N=10 000 (bottom row). The y‐axis and the vertical violin plots show the distributions of the estimates ${\hat{\beta }}_{F}$ with no uncertainty in the study weights (in red) and ${\hat{\hat{\beta }}}_{F}$ with estimated study weights (in gray). The x‐axis and the horizontal violin plot show the distribution of the estimated weight given to study 1 in ${\hat{\hat{\beta }}}_{F}$. Notice that this same x‐coordinate is used for both the gray and red diamonds, to illustrate their variability and their overlap (in the homogeneous case), but the weight for study 1 in ${\hat{\beta }}_{F}$ is always exactly 0.5, as indicated by the red line in the horizontal violin plot

Under exact homogeneity, the second term in Equation 6 simplifies to zero, but is otherwise strictly positive. Moreover, this second term does not become small compared with the first term at larger sample sizes. Before showing this phenomenon formally, we first illustrate it in Figure 1. It shows a simple fixed effects meta‐analysis of just 2 studies, of equal sample size, precision, but potentially with unequal *β*_*i*_. Comparing behavior of the fixed effects estimate with known standard errors ( ${\hat{\beta }}_{F}$, in red) and estimated standard errors ( ${\hat{\hat{\beta }}}_{F}$, in gray), we see that for heterogeneous data, regardless of sample size, the estimated standard errors give a more variable estimate. This is because ${\hat{\hat{\beta }}}_{F}$ is “tilted” closer to *β*_1_ or *β*_2_ when—by chance alone—study 1 or 2 receives greater weight. This pattern persists at larger sample sizes, so while the absolute amount of extra noise induced is reduced, the relative variabilities remain essentially unchanged. For the homogeneous settings, the 2 *β*_*i*_ are equal, so no “tilting” occurs, but for the heterogenous settings, the precisions differ by a factor of more than 5.

To build further intuition about the extra variability induced by using estimated standard errors, we now provide an analytic version of the results illustrated in Figure 1. To do this, we write the variance of each ${\hat{\beta }}_{i}$ as $\mathrm{Var}({\hat{\beta }}_{i})=\sigma_{i}^{2}={\left(n_{i}\phi_{i}\right)}^{-1}$, and the estimator $s_{i}^{2}$ of $\sigma_{i}^{2}$ as $s_{i}^{2}={\left(n_{i}{\hat{\phi }}_{i}\right)}^{-1}$, so that

(7) $${\hat{\hat{\beta }}}_{F}=\frac{\sum_{i}^{k}\frac{1}{s_{i}^{2}}{\hat{\beta }}_{i}}{\sum_{i}^{k}\frac{1}{s_{i}^{2}}}=\frac{\sum_{i}^{k}n_{i}{\hat{\phi }}_{i}{\hat{\beta }}_{i}}{\sum_{i}^{k}n_{i}{\hat{\phi }}_{i}}.$$

Additionally, we make the large‐sample approximation that each ${\hat{\phi }}_{i}$ is asymptotic normal, with asymptotic variance given by some function of the distributional moments of the population(s) in study *i*, that we write as *f*_*i*_(***θ***_*i*_). 1The specific form of *f*_*i*_(***θ***_*i*_) will depend on the type of estimator used, the study's randomization ratio as well as the variances and kurtoses of the treatment and control subpopulations. For this reason, we have decided to use this generic expression but have also provided detailed case‐specific derivations in Appendix E
. Using the usual assumptions of normality of ${\hat{\beta }}_{i}$ and independence of ${\hat{\beta }}_{i},s_{i}$, then by the delta method, we obtain

(8) $$\sqrt{N}\left({\hat{\hat{\beta }}}_{F}-\beta_{F}\right)\to_{d}N\left(0,\frac{1}{\left(\sum_{i}^{k}\eta_{i}\phi_{i}\right)}\left[1+\frac{\sum_{i}^{k}\eta_{i}{\left(\beta_{i}-\beta_{F}\right)}^{2}f_{i}(\theta_{i})}{\sum_{i}^{k}\eta_{i}\phi_{i}}\right]\right).$$

**Figure 2:**
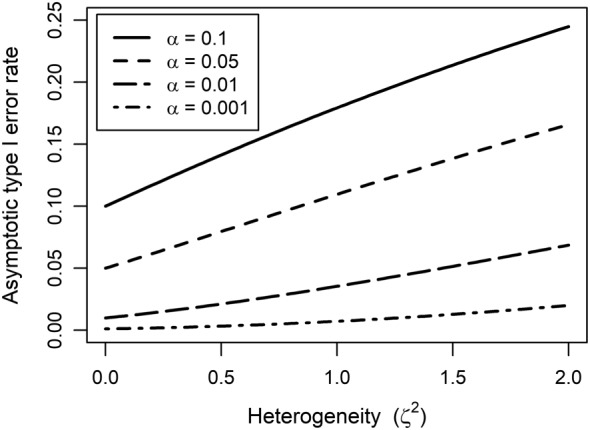
Inflated asymptotic type I error rate for the test of hypothesis H_0_:β_F_=0 in the presence of heterogeneity, when using a naive estimator of the variance from Equation 5 for a simple case of difference in means of continuous normal outcome with constant variance and balanced study designs (see details in Appendix E1)

**Figure 3:**
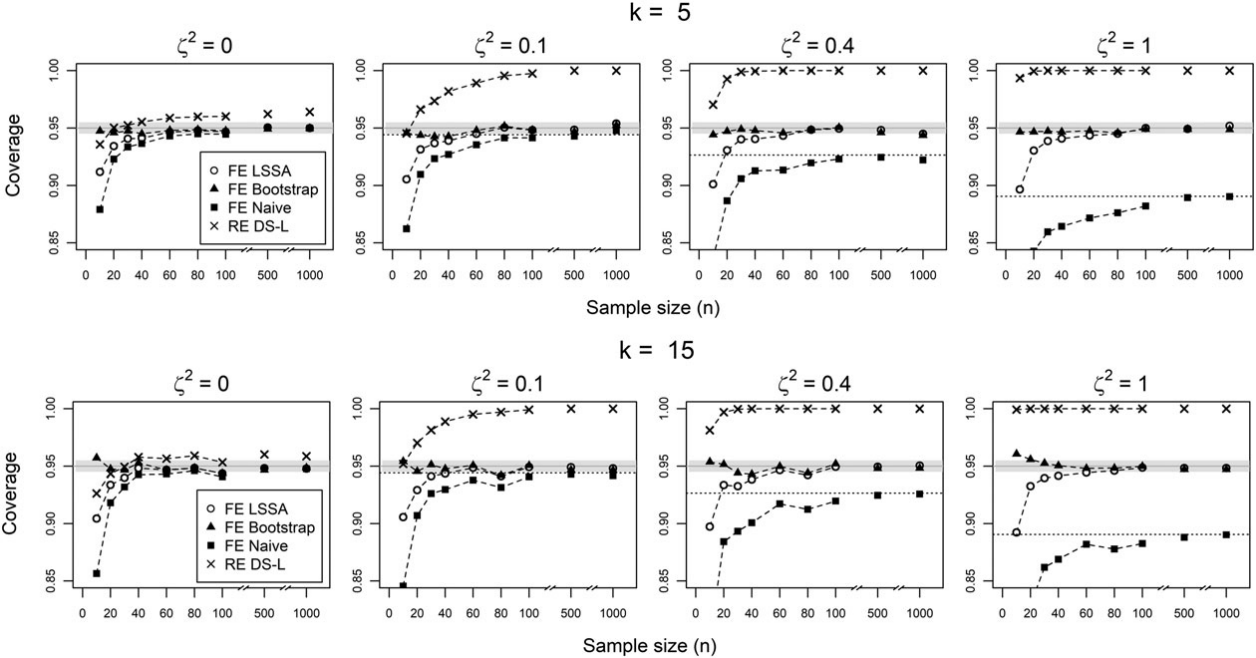
Coverage probabilities of 95% confidence intervals for β_F_=0 from 10 000 simulations, using the fixed effects approach large sample size approximation (LSSA) estimator for the variance and bootstrap percentiles, compared to the “naive” estimator from a common effect approach and the DerSimonian‐Laird estimator from a random effects approach

Details are provided in Appendix B. Comparing Equation 8 to the standard error in Equation 5, we see that the asymptotic variance of ${\hat{\hat{\beta }}}_{F}$ is the product of the asymptotic variance when the variances are known multiplied by an *inflation factor*, given in square brackets. This inflation factor, which accounts for the uncertainty in the estimation of the standard errors, depends on the squared deviations of *β*_*i*_ from *β*_*F*_, and thus, it will reduce to 1 under homogeneity but will increase as the dispersion of the effect sizes increases. We also notice that the squared deviations are multiplied by *f*_*i*_(***θ***_*i*_), the asymptotic variance of the information *ϕ*_*i*_, implying that the inflation factor increases when the studies are less informative about *ϕ*. Figure 2 illustrates, for a simple case, the nontrivial impact of inflation on type I error rates when testing a point null hypothesis for *β*_*F*_, even in large samples. The overstatements of statistical significance depend on the heterogeneity present, but also the nominal level *α*. (Full details are given in Appendices B and E1). This theoretic result underpinning Figure 2 has been confirmed empirically in a simulation study (Figure 3), described in Section 4.3.

### 3.3 A parameter to quantify heterogeneity

While quantifying heterogeneity in meta‐analyses has an obvious scientific appeal—describing how effects differ across study populations—the results of Section 3.2 do also suggest a statistical role for consideration of heterogeneity. Bridging these 2 goals, we now propose a parameter to quantify the heterogeneity of a group of effect‐size parameters.

As a natural extension of the location‐summary *β*_*F*_, we define

(9) $$\zeta^{2}=\frac{\sum_{i=1}^{k}\frac{1}{\sigma_{i}^{2}}{\left(\beta_{i}-\beta_{F}\right)}^{2}}{\sum_{i=1}^{k}\frac{1}{\sigma_{i}^{2}}}=\frac{\sum_{i=1}^{k}\frac{1}{\sigma_{i}^{2}}\beta_{i}^{2}}{\sum_{i=1}^{k}\frac{1}{\sigma_{i}^{2}}}-\beta_{F}^{2}=\frac{\sum_{i=1}^{k}\eta_{i}\phi_{i}\beta_{i}^{2}}{\sum_{i=1}^{k}\eta_{i}\phi_{i}}-\beta_{F}^{2},$$

where *β*_*F*_ is as in Equation 4. For fixed sample size proportions *η*_1_,…,*η*_*i*_, we can see that *ζ* ^2^ is also a population parameter, just like *β*_*F*_. We can interpret *ζ* ^2^ as a weighted average of the squared deviations of each study effect size from the weighted average effect *β*_*F*_, where the weights are proportional to the precision (or the proportion of information) associated with each study effect. Consequently, deviations from more precisely estimable study effects are upweighted. This parameter *ζ* ^2^ is a weighted average squared deviation and quantifies the heterogeneity of the effect sizes.

As shown in Appendix C, *ζ* ^2^ can also be defined without regard to *β*_*F*_, as a summary of pairwise comparisons of the *β*_*i*_, by writing it in the form

$$\zeta^{2}=\frac{1}{2}\frac{\sum_{i=1}^{k}\sum_{j=1}^{k}\frac{1}{\sigma_{i}^{2}}\frac{1}{\sigma_{j}^{2}}{\left(\beta_{i}-\beta_{j}\right)}^{2}}{{\left(\sum_{i=1}^{k}\frac{1}{\sigma_{i}^{2}}\right)}^{2}}=\frac{\sum_{1\leq i<j\leq k}\frac{1}{\sigma_{i}^{2}}\frac{1}{\sigma_{j}^{2}}{\left(\beta_{i}-\beta_{j}\right)}^{2}}{{\left(\sum_{i=1}^{k}\frac{1}{\sigma_{i}^{2}}\right)}^{2}}.$$

Specifically, *ζ* ^2^ is the weighted average of the pairwise differences of the effect sizes, weighting each pair by the product of their corresponding precisions. Unlike the between‐studies variance *τ* ^2^ used in random effects approaches, *ζ* ^2^ is defined on just the studies at hand, not a hypothetical population of potential studies, and some scheme for sampling from this population.

Although the definition of *ζ* ^2^ is free of distributional assumptions, it can further justified if we assume normality of the effect‐size estimators (see, eg, Rice et al^4^). Under this assumption, the *Q* statistic is distributed noncentral *χ* ^2^ with *k*−1 degrees of freedom and noncentrality parameter *λ* given by

(10) $$\lambda =\sum_{i=1}^{k}\frac{1}{\sigma_{i}^{2}}{\left(\beta_{i}-\beta_{F}\right)}^{2}=\left(\sum_{i=1}^{k}\frac{1}{\sigma_{i}^{2}}\right)\left(\frac{\sum_{i=1}^{k}\frac{1}{\sigma_{i}^{2}}{\left(\beta_{i}-\beta_{F}\right)}^{2}}{\sum_{i=1}^{k}\frac{1}{\sigma_{i}^{2}}}\right)=\left(\sum_{i=1}^{k}n_{i}\phi_{i}\right)\zeta^{2}=\Phi \zeta^{2},$$

where we have used $\Phi =\sum n_{i}\phi_{i}=N\sum \eta_{i}\phi_{i}$ to denote the total amount of information. This expression means that *λ*, and thus the power of the test of homogeneity based on *Q*, depends on 2 components: one is the total amount of information, which in turn depends on the total sample size, and the other is the heterogeneity between effect sizes, as given by *ζ* ^2^, which is independent of the total sample size. In other words, *ζ* ^2^ provides a measure of the distance from the null hypothesis of homogeneity.

## 4 INFERENCE FOR β_F_ AND ζ ^2^

### 4.1 Inference for β_F_ and ζ ^2^ with known standard errors

Inference for ${\hat{\beta }}_{F}$ with known standard errors was described in Equation 2; confidence intervals for *β*_*F*_ are usually built from a normal approximation, appealing to the large sample properties of ${\hat{\beta }}_{i}$. For a full description, see, eg, Hartung and Knapp.^6^

For the estimation of the heterogeneity parameter *ζ* ^2^, with known standard errors and efficient ${\hat{\beta }}_{i}$, we write $\sigma_{i}^{2}={\left(n_{i}\phi_{i}\right)}^{-1}$ for *i*=1,…,*k* and also define $\Phi =\sum_{i=1}^{k}n_{i}\phi_{i}$ as the “total information.” Then with no further distributional assumptions, a simple moment‐based point estimate of *ζ* ^2^ is given by

(11) $${\hat{\zeta }}^{2}=\frac{\sum_{i=1}^{k}\sigma_{i}^{-2}{\left({\hat{\beta }}_{i}-{\hat{\beta }}_{F}\right)}^{2}-(k-1)}{\sum_{i=1}^{k}\sigma_{i}^{-2}}=\frac{Q-(k-1)}{\Phi },$$

with details given in Appendix D. To give a strictly positive estimator of *ζ* ^2^, we can report

$${\hat{\zeta }}_{0}^{2}=\max\left(0,\frac{Q-(k-1)}{\Phi }\right).$$

To obtain approximate confidence intervals for *ζ* ^2^, we assume normality of the effect‐size estimators and exploit the relationship between *ζ* ^2^ and the noncentrality parameter *λ* as given in Equation 10. We proposed using methods for constructing exact confidence intervals for the noncentrality parameter of a chi‐square distribution that have been proposed and evaluated previously.^42^, ^43^ Basically, these methods consist on inverting a probability interval of the non‐central *χ* ^2^ distribution. For example, for given for given Φ and *Q*, a (1−*α*)×100% confidence interval for *ζ* ^2^ is given by all the values for which

$$\chi_{k-1,\alpha /2}^{2}(\Phi \zeta^{2})\leq Q\leq \chi_{k-1,1-\alpha /2}^{2}(\Phi \zeta^{2}).$$

Solutions can be obtained numerically, and code for this and other types of confidence intervals (for the noncentrality parameter) is available.^43^

### 4.2 Inference on for β_F_ and ζ ^2^ with estimated standard errors

#### 4.2.1 Large sample size approximation

Based on Equation 8, a large sample size approximation (LSSA) of the variance of ${\hat{\hat{\beta }}}_{F}$ is given by

(12) $$\mathrm{Var}[{\hat{\hat{\beta }}}_{F}]\approx \frac{1}{N\left(\sum_{i}^{k}\eta_{i}\phi_{i}\right)}\left[1+\frac{\sum_{i}^{k}\eta_{i}{\left(\beta_{i}-\beta_{F}\right)}^{2}f_{i}(\theta_{i})}{\sum_{i}^{k}\eta_{i}\phi_{i}}\right].$$

Further details on the specific form of *f*_*i*_(***θ***_*i*_) in (12) for some common effect‐size estimators are provided in Appendix E. In situations where the function *f*_*i*_ is known or can be estimated, tests of hypothesis and confidence intervals can be based on a normal approximation using a plug‐in estimator of (12) with the estimates of *β*_*i*_,*ϕ*_*i*_ and ***θ***_*i*_ for 1≤*i*≤*k* and *β*_*F*_. The (1−*α*)×100*%* LSSA interval then takes the form

$${\hat{\hat{\beta }}}_{F}\pm z_{1-\alpha /2}\frac{1}{\sqrt{\sum_{i=1}^{k}n_{i}{\hat{\phi }}_{i}}}{\left[1+\frac{\sum_{i}^{k}n_{i}{\left({\hat{\beta }}_{i}-{\hat{\hat{\beta }}}_{F}\right)}^{2}f_{i}({\hat{\theta }}_{i})}{\sum_{i}^{k}n_{i}{\hat{\phi }}_{i}}\right]}^{1/2}.$$

#### 4.2.2 Quasi‐F approach

We next propose an interval based on inverting a test of the null hypothesis of homogeneity, similarly to Hartung and Knapp.^5^ It is based on a “quasi‐F” test statistic, a statistic that approximates a *F*‐distributed random variable.^44^

To construct it, we use normality of the ${\hat{\beta }}_{i}$ to provide

$$\begin{array}{ccc} \frac{{\left({\hat{\beta }}_{F}-\beta_{F0}\right)}^{2}}{\mathrm{Var}[{\hat{\beta }}_{F}]} & ∼\chi_{1}^{2}, & \\ Q & ∼\chi_{k-1}^{2}(\lambda ),\;\text{with}\;\lambda =\sum_{i=1}^{k}n_{i}\phi_{i}{\left(\beta_{i}-\beta_{F}\right)}^{2}=\Phi \zeta^{2}, \end{array}$$

for null value *β*_*F*0_. Approximating the noncentral *χ* ^2^ distribution by matching its moments to a central *χ* ^2^,^45^, ^46^ we can approximate the distribution of *Q* as a *α*‐scaled central *χ* ^2^ distribution with *ν* degrees of freedom ( $\alpha \chi_{\nu }^{2}$), where

$$\begin{array}{ccc} \alpha & =1+\frac{\lambda }{(k-1)+\lambda }=1+\frac{\Phi \zeta^{2}}{(k-1)+\Phi \zeta^{2}} & \\ \nu & =(k-1)+\frac{\lambda^{2}}{(k-1)+2\lambda }=(k-1)+\frac{{\left(\Phi \zeta^{2}\right)}^{2}}{(k-1)+2\Phi \zeta^{2}}. \end{array}$$

Under the assumptions above, *Q* and ${\hat{\beta }}_{F}$ are independent,^4^, ^5^ so

$$\frac{{\left({\hat{\beta }}_{F}-\beta_{0}\right)}^{2}/\mathrm{Var}[{\hat{\beta }}_{F}]}{Q/\alpha \nu }$$

has an approximate $F_{\nu }^{1}$ distribution, and its signed square root has an approximate Student *t* distribution with *ν* degrees of freedom.

To use these results with unknown *σ*_*i*_, a “quasi‐F” statistic can be constructing by plugging‐in estimators of all those quantities. Thus, letting $\hat{\hat{\beta }}$ be as in Equation 5, $\hat{\mathrm{Var}}[{\hat{\hat{\beta }}}_{F}]$ the LSSA given in Equation 12, along with plug‐in estimates of *Q*, *ζ* ^2^, and ϕ, used in turn to estimate *α* and *ν*. Taking square roots, the test statistic

(13) $$t=\sqrt{\frac{\hat{\alpha }\hat{\nu }{\left({\hat{\hat{\beta }}}_{F}-\beta_{F0}\right)}^{2}}{\hat{\mathrm{Var}}[{\hat{\hat{\beta }}}_{F}]\hat{Q}}}$$

has an approximate Student *t* distribution with $\hat{\nu }$ degrees of freedom under the null hypothesis *H*_0_:*β*_*F*_=*β*_*F*0_. (This reference distribution would differ importantly from a standard normal for small values of $\hat{\nu }$, which would be expected when the meta‐analysis includes few studies (small *k*) and the total amount of information times the amount of heterogeneity is small, ie, approaching the limit where ϕ*ζ* ^2^→0.) Inverting this test, we obtain an approximate confidence interval for *β*_*F*_.

#### 4.2.3 Parametric bootstrap

The alternative estimators described in Sections 4.2.1 and 4.2.2, which take into account the potential heterogeneity of the effect‐size parameters, are based on approximations that would be expected to work in large sample settings, but would probably perform poorly in settings with very small size samples. An alternative method that could better in small sample size settings is bootstrap re‐sampling. As individual‐level observations are typically not available, we consider using parametric bootstrap sampling. (For a full review of this approach, see chapter 6 of Efron and Tibshirani^47^)

Estimates of the variance of ${\hat{\hat{\beta }}}_{F}$, as well as 95% confidence intervals, and/or *P* values for testing of hypothesis can all be obtained from parametric sampling, based on the estimates ${\hat{\beta }}_{1},{\hat{\beta }}_{2},\text{⋯},{\hat{\beta }}_{k}$ and $s_{1}^{2},s_{2}^{2},\text{⋯},s_{k}^{2}$. Assuming a normal distribution of the effect sizes estimates, a parametric bootstrap sample of size *B* for each of the effect‐size parameters *β*_*i*_ can be obtained:

(14) $${\hat{\beta }}_{i[b]}^{*}∼N({\hat{\beta }}_{i},s_{i}^{2}),\;\text{for}\;i=1,\text{⋯},k;b=1,\text{⋯},B.$$

However, parametric sampling for the variances of the effect estimates depends on the specific variance estimator used in each study. For example, for the variance of the difference in means of independent groups where equal variances are assumed, a bootstrap sample of ${\hat{\sigma }}_{i}^{2}$ can be obtained as

(15) $${\hat{\sigma }}_{i[b]}^{2*}=\frac{{\hat{\varsigma }}_{i[b]}^{*2}}{n_{i}}\;\;\text{with}\;{\hat{\varsigma }}_{i[b]}^{2*}∼\frac{{\hat{\varsigma_{i}}}^{2}}{n_{i}-2}\chi_{n_{i}-2}^{2},\;\text{for}\;i=1,\text{⋯},k;b=1,\text{⋯},B,$$

where ${\hat{\varsigma }}_{i}^{2}$ is the pooled estimate of the common variance $\varsigma_{i}^{2}$.^48^ More generally, for estimates from linear regression (where normality and constant variance are assumed), the sampling can be done from a *χ* ^2^ distribution with (*n*_*i*_−*p*_*i*_) degrees of freedom, where *p*_*i*_ denotes the number of predictors in the regression (including the intercept). In contrast, when *β*_*i*_ is estimated as the difference in means of independent groups with the variances not assumed to be equal, the parametric sampling of $\varsigma_{i,X}^{2}$ and $\varsigma_{i,Y}^{2}$ should be done separately and then combined to obtain the value of $\sigma_{i}^{2}$. Further details on the specific form of some of these estimators can found in Appendix E.

From the parametric bootstrap samples of effect size and variance estimators, different estimates and/or test statistics can be obtained. We propose (and evaluate) the following:

1. A pivotal (1−*α*)% confidence interval based on a normal approximation and using an estimate of the variance of ${\hat{\hat{\beta }}}_{F}$ from a bootstrap sample (see chapter 6 of Efron and Tibshirani^47^):
  $${\hat{\hat{\beta }}}_{F}\pm z_{1-\alpha /2}\sqrt{\frac{1}{B-1}\sum_{b=1}^{B}{\left({\hat{\beta }}_{F[b]}^{*}-\frac{1}{B}\sum_{b=1}^{B}{\hat{\beta }}_{F[b]}^{*}\;\right)}^{2}},\;\text{where}\;{\hat{\beta }}_{F[b]}^{*}=\frac{\sum_{i=1}^{k}\frac{1}{{\hat{\sigma }}_{i[b]}^{2*}}{\hat{\beta }}_{i[b]}^{*}}{\sum_{i=1}^{k}\frac{1}{{\hat{\sigma }}_{i[b]}^{2*}}}$$
2. A (1−*α*)% confidence interval constructed from the percentiles of the empirical distribution of the bootstrap sample of ${\hat{\beta }}_{F[b]}^{*}$, as defined in (1), (see chapter 13 of Efron and Tibshirani^47^):
  $$\left({\hat{\beta }}_{F(\alpha /2)}^{*},{\hat{\beta }}_{F(1-\alpha /2)}^{*}\right).$$
3. A Bootstrap‐*t* confidence interval (see chapter 12 of Efron and Tibshirani^47^), based on the percentiles from the distribution of a test statistic constructed using a “naive” estimator of the variance of ${\hat{\hat{\beta }}}_{F}$:
  $$\left({\hat{\hat{\beta }}}_{F}-t_{\left(1-\alpha /2\right)}^{*}\sqrt{{\left(\sum_{i=1}^{k}\frac{1}{s_{i}^{2}}\right)}^{-1}},{\hat{\hat{\beta }}}_{F}-t_{\left(\alpha /2\right)}^{*}\sqrt{{\left(\sum_{i=1}^{k}\frac{1}{s_{i}^{2}}\right)}^{-1}}\right),\;\text{where}\;t_{\left[b\right]}^{*}=\frac{{\hat{\beta }}_{F[b]}^{*}}{\sqrt{{\left(\sum_{i=1}^{k}\frac{1}{{\hat{\sigma }}_{i[b]}^{2*}}\right)}^{-1}.}}$$
4. A Bootstrap‐*t* confidence interval, based on the percentiles from the distribution of a test statistic constructed using the LSSA estimator of the variance of ${\hat{\hat{\beta }}}_{F}$, as given in (12):
  $$\left({\hat{\hat{\beta }}}_{F}-t_{\left(1-\alpha /2\right)}^{*}\sqrt{\hat{\mathrm{Var}}[{\hat{\hat{\beta }}}_{F}]},{\hat{\hat{\beta }}}_{F}-t_{\left(\alpha /2\right)}^{*}\sqrt{\hat{\mathrm{Var}}[{\hat{\hat{\beta }}}_{F}]}\right),\;\text{where}\;t_{\left[b\right]}^{*}=\frac{{\hat{\beta }}_{F[b]}^{*}}{\sqrt{{\mathrm{Var}}_{\left[b\right]}^{*}[{\hat{\hat{\beta }}}_{F}]}}.$$

Similar approaches are proposed for the heterogeneity parameter *ζ* ^2^, based on a bootstrap sample of the estimator proposed in (14) and (15)

$${\hat{\zeta }}_{\left[b\right]}^{2*}=\frac{\sum_{i=1}^{k}{\hat{\sigma }}_{i[b]}^{-2*}{\left({\hat{\beta }}_{i[b]}^{*}-{\hat{\beta }}_{F[b]}^{*}\right)}^{2}-(k-1)}{\sum_{i=1}^{k}{\hat{\sigma }}_{i[b]}^{-2*}},\;\text{for}\;b=1,\text{⋯},B.$$

We present evaluations of the coverage of confidence intervals using 2 approaches (some other alternatives were attempted, but did not show important improvement):

1. A pivotal (1−*α*)% confidence interval based on a normal approximation and using an estimate of the variance of ${\hat{\zeta }}^{2}$ from the bootstrap sample:
  $${\hat{\zeta }}^{2}\pm z_{1-\alpha /2}\sqrt{\frac{1}{B-1}\sum_{b=1}^{B}{\left({\hat{\zeta }}_{\left[b\right]}^{2*}-\frac{1}{B}\sum_{b=1}^{B}{\hat{\zeta }}_{\left[b\right]}^{2*}\;\right)}^{2}},$$
  where ${\hat{\zeta }}_{\left[b\right]}^{2*}$ is defined as above.
2. A (1−*α*)% confidence interval constructed from the percentiles of the empirical distribution of the bootstrap sample of ${\hat{\zeta }}_{\left[b\right]}^{2*}$:
  $$\left({\hat{\zeta }}_{\left(\alpha /2\right)}^{2*},{\hat{\zeta }}_{\left(1-\alpha /2\right)}^{2*}\right).$$

### 4.3 Simulation study

We conducted a simulation study to evaluate and compare the different estimation methods proposed for *β*_*F*_ and *ζ* ^2^. For our simulations, we considered fixed effect sizes (*β*_1_,…,*β*_*k*_), uniformly spaced and centered around zero (*β*_*F*_=0), with the spacing in between given by fixed values of *ζ* ^2^. We assumed continuous normal outcomes and the effect size *β*_*i*_ given by the mean difference between 2 groups, assuming equal population variances and balanced designs. We took random draws of the effect estimates ( ${\hat{\beta }}_{1},\text{⋯},{\hat{\beta }}_{k}$) from normal distributions centered around the fixed effects (*β*_1_,…,*β*_*k*_) along with random draws of their variances taken from scaled *χ* ^2^ distributions with *n*_*i*_−2 degrees of freedom. Various scenarios were considered, varying the number of studies, sample sizes, and amount of heterogeneity. In addition to the various confidence intervals proposed here for *β*_*F*_ and *ζ* ^2^, we also compared their performance with methods typically used in meta‐analysis, ie, the common effect and random effects approaches. To aid the comparisons, we chose a setup in which all these approaches estimate location parameters with the same numerical value. Further details on the settings and complete results from the simulation study can be found in the supporting information for the online article.

Representative results are shown in Figure 3. For the estimation of the location parameter *β*_*F*_, we observed a better performance of parametric bootstrap methods over those based on asymptotic approximations, especially with small sample sizes. Among these, the confidence interval based on the percentiles of the empirical distribution of the parametric sample would be recommended, because it is simple and performed well, providing coverage close to nominal level. However, we also notice that the LSSA method performed reasonably well for large sample sizes (at least 60 subjects per study) and note that it can be used if the parametric bootstrap could not be implemented.

Compared to existing methods, as expected, the random effects approach (using the DerSimonian‐Laird estimator of *μ*) provided overconservative inference, as result of wide confidence intervals that account for random sampling of effect sizes that is not present in our simulation settings. However, for the common effect estimator, which is equivalent to use a naïve estimate of the variance of *β*_*F*_ as given in Equation 7, the coverage probability approaches the nominal level as the sample size increases but never reaching it in the presence of heterogeneity. The asymptotic coverage of this naive estimator has been calculated using (12) and is shown as dotted horizontal lines in Figure 3 (see details in Appendix E1).

**Figure 4:**
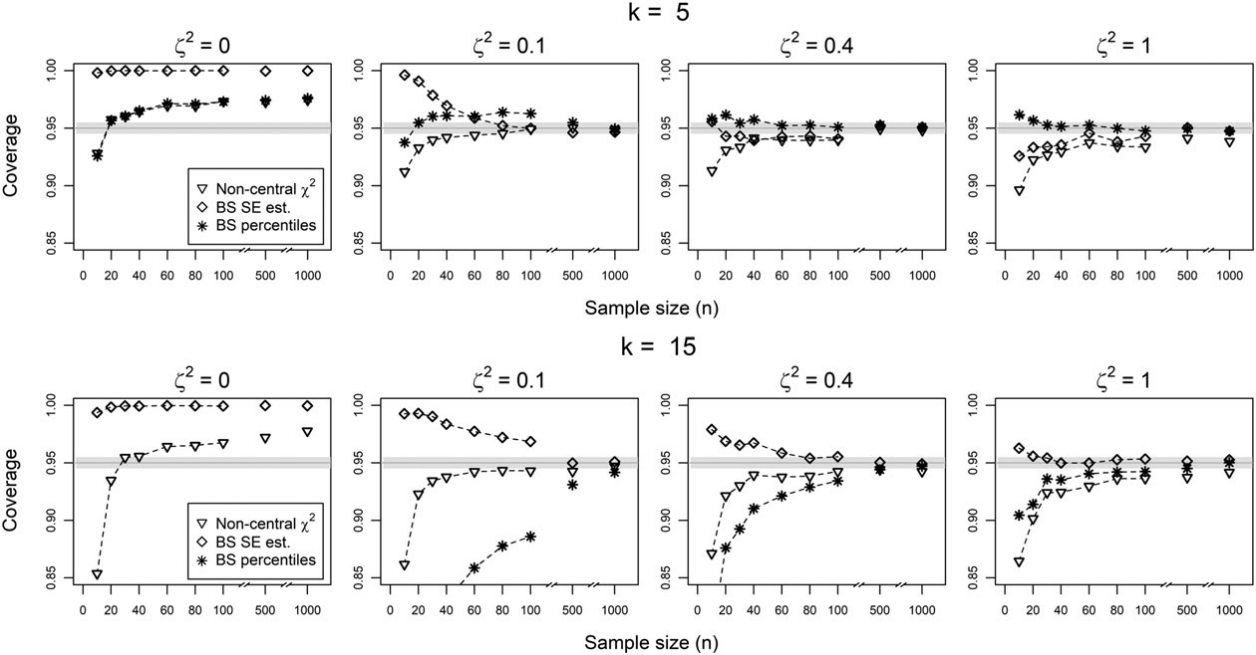
Coverage probabilities of 95% confidence intervals for ζ ^2^ from 10 000 simulations, using an inverted probability interval from a noncentral χ ^2^ distribution, a normal approximation with bootstrap estimate of the standard error and a bootstrap estimate based on the quartiles of the empirical distribution

For the heterogeneity parameter *ζ* ^2^, although all the proposed methods seemed to asymptotically achieve the nominal coverage probability, none of them performed uniformly better for small sample size settings in all scenarios (Figure 4). The normal approximation with a moment based estimate of the standard error showed both significant overcoverage and undercoverage in different scenarios (not shown). The normal approximation using a Bootstrap estimate of the standard error seemed to correct the undercoverage in some scenarios, but not when the number of studies was small (*k*=3), while the bootstrap confidence intervals based on the percentiles showed important undercoverage for low values of heterogeneity and large number of studies (*k*=7,15). This result is consistent with a previous result, in which the consistency of bootstrap estimation is related to the asymptotic normality of the statistic,^49^, ^50^ while in our case, distribution of the statistic is far from normal, for small sample size and low level of heterogeneity. On the other hand, given the more consistent performance of the inverted probability interval from a noncentral *χ* ^2^ distribution, we would recommend its use when the sample sizes are large enough (at least 40 observations per study) and the studies are not strongly heterogeneous.

## 5 EXAMPLE

**Figure 5:**
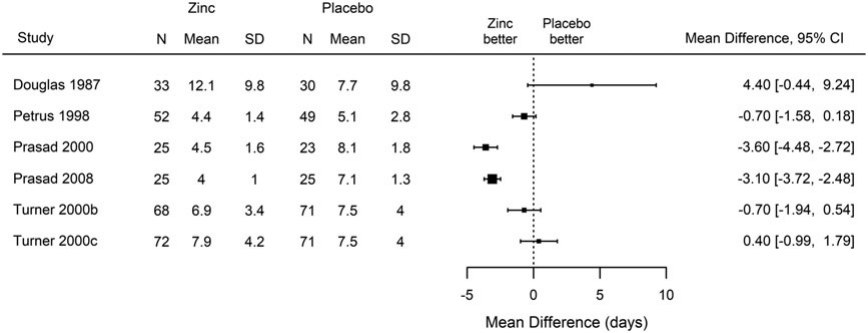
Meta‐analysis on the efficacy of zinc acetate lozenges in reducing the duration of cold symptoms^51^

In this section, we apply the estimation methods discussed in Section 4 to an example from a systematic review of studies that evaluate the efficacy of zinc in reducing the incidence, severity, and duration of common cold symptoms.^51^ In this particular meta‐analysis, the authors included studies that compare zinc acetate lozenges with placebo, with the outcome being the duration of cold symptoms (in days) and the treatment effect measured by the mean difference. A forest plot is shown in Figure 5.

In Table 2, we summarize the results of meta‐analyses on the 6 studies comparing zinc lozenges to placebo, using 3 different approaches. We observe that the point estimates of *β*_0_ and *β*_*F*_ from the common effect and fixed effects approaches, respectively, although numerically the same ( ${\hat{\beta }}_{0}={\hat{\beta }}_{F}=-2.04$ days), estimate different parameters. The first estimates a common effect underlying all 6 studies, but given the evident heterogeneity between studies, this inference does not seem to be adequate, or even valid. On the other hand, ${\hat{\beta }}_{F}$ estimates a weighted average of the mean differences from the 6 studies, for which a significant amount of heterogeneity is observed, as reflected by the estimate of *ζ* ^2^. More specifically, ${\hat{\beta }}_{F}$ estimates the mean difference in duration of common cold averaged in a meta‐population composed of the populations from which the samples of these 6 studies were drawn, in proportions given by $\sigma_{i}^{-2}/\sum_{i}^{k}\sigma_{i}^{-2}$. Similarly, *ζ* ^2^ can be thought as estimating how far apart the mean differences in 2 of these populations are, averaged over the same meta‐population. We also observe that the results from different estimation methods, although not exactly the same, do not seem to differ importantly, with a difference in length of 0.13 days between the 95% confidence intervals using the LSSA and the parametric bootstrap.

**Table 2:** Fixed and random effects approaches to the meta‐analyses on the effect of zinc acetate lozenges, estimated as the mean difference in the duration of symptoms of the common cold (in days),^51^ with point estimates and 95% confidence intervals obtained from different methods of estimation

Common effect	${\hat{\beta }}_{0}$ (95% CI)
	−2.04 (−2.45, −1.64)
Fixed effects	${\hat{\beta }}_{F}$ (95% CI)	$\hat{\zeta^{2}}$ (95% CI)
Assuming known *σ*_*i*_
Naive estimator	−2.04 (−2.45, −1.64)	
Noncentral *χ* ^2^ inverted test		2.09 (1.09, 3.50)
Unknown *σ*_*i*_
Large sample size approximation (LSSA)	−2.04 (−2.51, −1.57)
Quasi‐F–based Student *t*	−2.04 (−2.53, −1.55)
Parametric bootstrap (B = 2000)	−2.04 (−2.54, −1.59)	2.09 (1.15, 3.68)
Random effects	$\hat{\mu }$ (95% CI)	${\hat{\tau }}^{2}$ (95% CI)
DerSimonian‐Laird	−1.21 (−2.69, 0.28)	2.81 (1.19, 46.0)
Maximum likelihood	−1.21 (−2.69, 0.28)	2.79 (0, 6.53)
Restricted maximum likelihood	−1.13 (−2.83, 0.57)	3.78 (0, 9.25)
Sidik‐Jonkman	−1.02 (−3.06, 1.01)	5.66 (2.20, 34.04)

On the other hand, random effects meta‐analysis estimates the mean and variance of a population from which the effects in the 6 studies are thought to have been drawn (*μ* and *τ* ^2^). The inference now is not made for the population of subjects (on whom we wish to estimate an average effect of a treatment) but for a population of potential treatment effects. As shown in Table 2, different methods for estimating the between‐studies variance give notably different results, with larger estimates of *τ* ^2^ yielding estimates of *μ* that are closer to the unweighted simple average of the study effects (−0.56). Moreover, the precision with which these parameters are estimated is much smaller than the precision with which *β*_*F*_ and *ζ* ^2^ are estimated, even after taking into account the uncertainty in the estimation of the variances. This gain in precision, it should be noted, is not a result of a particular choice of estimation technique, it is instead the result of targeting our inference to a parameter that is easier to estimate, ie, one for which the the data provide most information.

**Figure 6:**
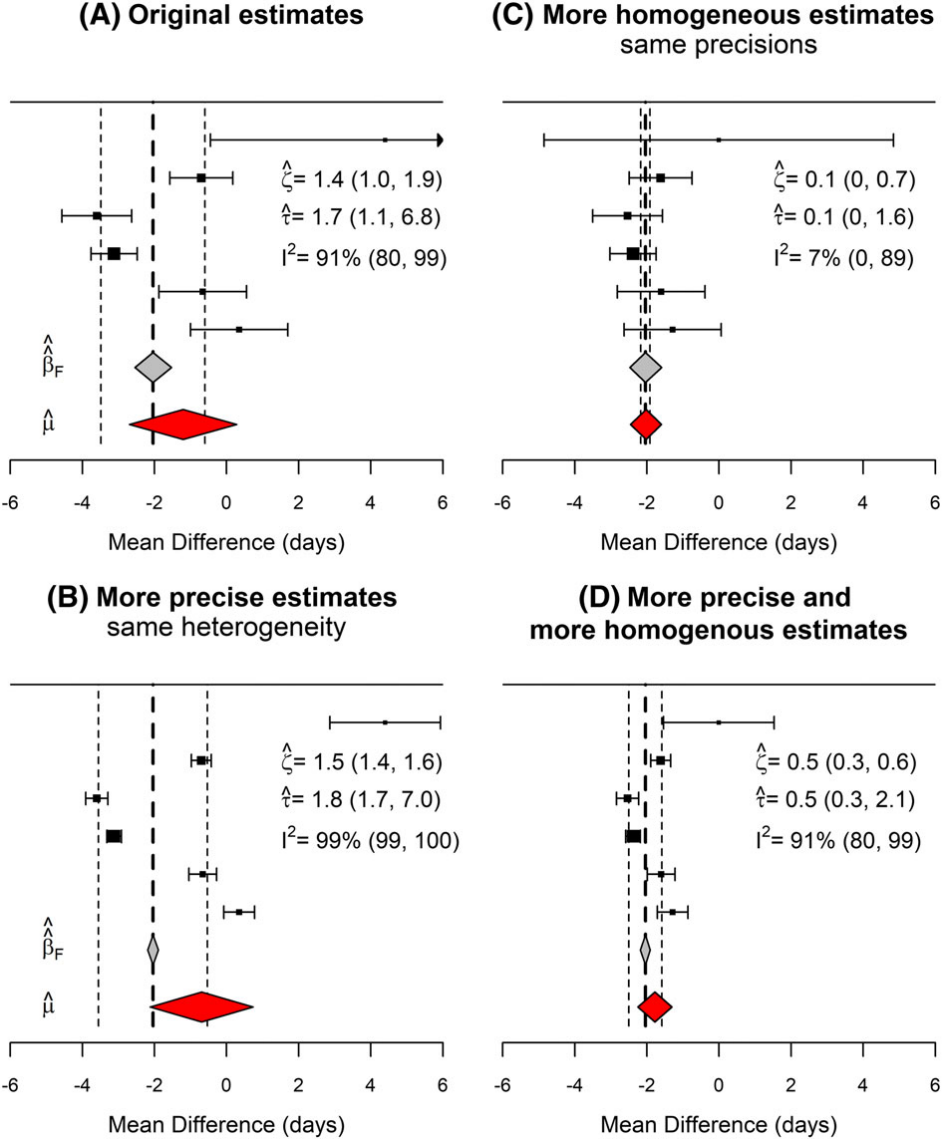
Location and scale parameter estimates for 4 different versions of the meta‐analysis in Figure 5: A, original estimates; B, more homogeneous estimates with squared deviation from ${\hat{\hat{\beta }}}_{F}$ reduced by a factor of 10; C, more precise estimates with sample sizes 10 times those of the original estimates; D, more precise and more homogeneous estimates, with sample sizes 10 times larger and squared deviations from ${\hat{\hat{\beta }}}_{F}$ reduced by a factor of 10, relative to the original estimates. Rhomboids are used to represent point estimates and 95% confidence intervals of location parameters β_F_ (in gray) and μ (in red). The vertical dashed lines represent the square root of the estimated averaged squared deviations from β_F_, as given by $\hat{\zeta }=\sqrt{\hat{\zeta^{2}}}$

To further illustrate the properties of the estimators of *β*_*F*_ and *ζ* ^2^ in a fixed effects meta‐analysis, we have modified the example into 3 different versions, as shown in Figure 6. First, we increased the precisions of the estimates in the meta‐analysis, by artificially growing the sample sizes by a factor of 10 (panel B). This results in a greater precision for the estimates of *β* and *ζ* ^2^. However, this same increase in information does not translate into an increased precision for estimating *μ* or *τ* ^2^ in a random‐effects model (for which more studies, rather than larger sample sizes, would be needed). In another version of the meta‐analysis, we have kept the same precision but shrunk (shifted) the estimates towards ${\hat{\beta }}_{F}$, so that the squared deviations have been reduced by factor of 10 (panel C). Reflecting this relative homogeneity, the estimate of *ζ* ^2^ is much lower and close to zero. We also notice that estimate of *β* remains practically unchanged, ie, is mostly independent of ${\hat{\zeta }}^{2}$(except for the variance inflation effect described in Section 3.2, which is not substantial in this case). In contrast, the estimate of *μ*, both in terms of its location and precision, is highly dependent on between study variance, as estimated by ${\hat{\tau }}^{2}$. Lastly, we artificially reduced the between‐study heterogeneity and the within‐study variance by the same factor (panel D). As a result of this, the value of the *Q* statistic is exactly the same as in the original version of the meta‐analysis (*Q* = 53.8, 5 degrees of freedom, *P* value <.0001), and so is the value for *I* ^2^. This makes sense, as in both meta‐analyses, heterogeneity accounts for the same proportion of the total variation. However, in absolute terms, the estimates in the modified version are much closer to each other than in the original meta‐analysis, and this is picked up by estimates of *ζ* ^2^ and *τ* ^2^, as they are both quantify “absolute” heterogeneity. Their confidence intervals in both cases exclude zero, rejecting the null hypothesis of homogeneous effects. However, as pointed out before, we can estimate *ζ* ^2^ with higher precision, even with few studies.

## 6 DISCUSSION

In this paper, we have addressed several aspects of the fixed effects meta‐analysis with within‐study estimates of the standard errors. To formally motivate its precision weighting, we described the optimality of the corresponding parameter *β*_*F*_, and by studying the behavior of the precision‐weighted estimate in detail, we showed the important role of a particular measure of heterogeneity, *ζ* ^2^.

Frequentist methods for the estimation of both the location parameter *β*_*F*_ and the heterogeneity parameter *ζ* ^2^ were proposed, including corrected estimators that take into account the uncertainty in the estimation of the within study variances. Estimation methods based on asymptotic approximations, as well as methods based on parametric bootstrap, were implemented and have been evaluated in a simulation study.

In the results of our simulation study, we observed a better performance of parametric bootstrap methods over those based on asymptotic approximations for the estimation of the location parameter *β*_*F*_, specially in small sample size settings. Among these, the confidence interval based on the percentiles of the empirical distribution of the parametric sample would be recommended, because of its simplicity and good performance. However, we also notice that the LSSA method performed reasonably well for large sample sizes (*n*≥60, per study) and could be used if the parametric bootstrap can not be implemented.

For the heterogeneity parameter *ζ* ^2^, although no method performed uniformly better, the construction of 95% confidence intervals by inverting the probability interval from a noncentral *χ* ^2^ distribution seems to provide close to nominal coverage when the sample size is large enough (around 40 observations per study).

The main limitation in our simulation study is that the proposed methods were implemented with knowledge of how the study estimates (including standard errors) were generated. The independence of the point estimate and standard errors—plausible in most uses of linear regression—may not be as realistic if the study‐specific analyses use logistic regression, or other forms of analysis under strong mean‐variance relationships. The normality of the ${\hat{\beta }}_{i}$ may also be considered a limitation, but unless the outcome variable is very heavy‐tailed and/or sensitive to a few observations, standard central limit theorem arguments suggest that this will only be an issue in extremely small samples.

We also illustrated the results of different estimation methods, as well as different approaches, with a previously published meta‐analysis. This example, along with the results of our simulation study, supports the idea of approaching meta‐analysis under a fixed effects framework, as a valid alternative to the typically used common effect and random effect approaches. Our approach, based on the estimation of both a location and a heterogeneity parameter, is more flexible than the restrictive common effect approach while allowing inference on the population of interest. Our approach also makes it unnecessary to choose between statistical models based on their adequacy rather than the target inference.

Finally, although we believe that estimation of both *β*_*F*_ and *ζ* ^2^ is useful for describing and combining in a meaningful way the effects of studies included in a meta‐analysis, we propose their estimation only as part of a full battery of qualitative and quantitative tools that should be used to review, summarize, and synthesize a group of studies. No single parameter or estimator can always appropriately summarize all there is to say in a systematic review of medical studies, and practitioners should be encouraged and helped to understand the measures they choose to provide.

## Supporting information



Coverage probability of 95% confidence intervals for *β*_*F*_ using (a) a ‘naive’ estimator of the standard error, (b) the large sample size approximation (LSSA) estimator of the standard error and (c) the t‐statistic from the quasi‐F approach, along with the DerSimonian‐Laird estimator for the mean of random effects. The dotted horizontal line represents the asymptotic coverage for the ‘naive’ estimator, calculated analytically (see Appendix E.1). These results are from 10,000 simulations, with the gray bar re ecting the approximate Monte Carlo error. See detailed description of these estimators in Sections 4.1 and 4.2.1 of the main paper.
Coverage probability of 95% confidence intervals for *β*_*F*_ obtained from parametric bootstrap samples of size 1000: (a) a normal approximation using an empirical estimate of standard error, (b) the percentiles of the empirical distribution, (c) Bootstrap‐*t* based on a *t*‐statistic using a naive estimation of the standard error and (d) Bootstrap‐*t* based on a *t*‐statistic using the LSSA estimation of the standard error. These results are from 10,000 simulations, with the grey bar reflecting the expected Monte Carlo error. See detailed description of these estimators in Section 4.3 of the main paper.
Coverage probability of 95% confidence intervals for ζ^2^ using: (a) an inverted probability interval from a non‐central χ^2^ distribution, (b) a normal approximation using an empirical estimate of the standard error from a parametric Bootstrap sample of size 1,000 and (c) the percentiles of the empirical distribution from the same parametric Bootstrap sample. These results are from 10,000 simulations, with the grey bar reflecting the expected Monte Carlo error. See detailed description of these estimators in Sections 4.1 to 4.3 of the main paper.

## APPENDIX A PROOF OF LEMMA 1

### A.1

Let ***v*** ^*T*^=(*v*_1_,*v*_2_,…,*v*_*k*_) be a vector of arbitrary weights with $\sum_{i=1}^{k}v_{i}=1$, and let $v^{T}\hat{\beta }=\sum v_{i}{\hat{\beta }}_{i}$ be the estimator of $v^{T}\beta =\sum v_{i}\beta_{i}$, with $\text{Cov}(\hat{\beta })=\Sigma$, then

$$\mathrm{Var}(v^{T}\hat{\beta })=v^{T}\Sigma v.$$

To minimize this expression, we use Lagrange multipliers:

$$\begin{array}{ccc} & \frac{d}{dv}\left[v^{T}\Sigma v+\lambda (1-v^{T}1)\right]=2\Sigma v-\lambda 1 & \\ & \frac{d}{d\lambda }\left[v^{T}\Sigma v+\lambda (1-v^{T}1)\right]=1-v^{T}1\\ & \Rightarrow v=\frac{\lambda }{2}\Sigma^{-1}1\text{;}\;\lambda =\frac{2}{1^{T}\Sigma^{-1}1} \end{array}$$

so then

$$w=\underset{v:v^{T}1=1}{\text{argmin}\;}\left[v^{T}\Sigma v\right]=\frac{\Sigma^{-1}1_{k}}{1_{k}^{T}\Sigma^{-1}1_{k}}$$

with

$$\mathrm{Var}(w^{T}\hat{\beta })=\frac{1_{k}^{T}\Sigma^{-1}\Sigma \Sigma^{-1}1_{k}}{{\left(1_{k}^{T}\Sigma^{-1}1_{k}\right)}^{2}}=\frac{1}{1_{k}^{T}\Sigma^{-1}1_{k}}.$$

## APPENDIX B DERIVATION OF THE ASYMPTOTIC VARIANCE OF ${\hat{\hat{\beta }}}_{F}$ (SECTION 3.2)

### B.1

First, we write $s_{i}^{2}={\left(n_{i}\phi_{i}\right)}^{-1}$ as the estimator of $\sigma_{i}^{2}={\left(n_{i}\phi_{i}\right)}^{-1}$, the variance of ${\hat{\beta }}_{i}$ in the *i* ^*t**h*^ study. We start by assuming that ${\hat{\beta }}_{i}$ and ${\hat{\phi }}_{i}$ are independent and have an asymptotic normal distribution:

(B1) $$\sqrt{n_{i}}\left(\left(\begin{array}{c} {\hat{\beta }}_{i}\\ {\hat{\phi }}_{i} \end{array}\right)-\left(\begin{array}{c} \beta_{i}\\ \phi_{i} \end{array}\right)\right)\overset{\;d\;}{\underset{}{\to }}N\left(\left(\begin{array}{c} 0\\ 0 \end{array}\right),\left(\begin{array}{cc} \phi_{i}^{-1} & 0\\ 0 & f_{i}(\theta_{i}) \end{array}\right)\right),$$

where *f*_*i*_(***θ***_*i*_) is some function of the distributional moments of the population(s) in study *i*. For a meta‐analysis of studies with different sample sizes, we define *η*_*i*_=*n*_*i*_/*N*, with $N=\sum_{i}^{k}n_{i}$. Then, dividing (B1) by $\sqrt{\eta_{i}}$, we get

$$\sqrt{N}\left(\left(\begin{array}{c} {\hat{\beta }}_{i}\\ {\hat{\phi }}_{i} \end{array}\right)-\left(\begin{array}{c} \beta_{i}\\ \phi_{i} \end{array}\right)\right)\overset{\;d\;}{\underset{}{\to }}N\left(\left(\begin{array}{c} 0\\ 0 \end{array}\right),\left(\begin{array}{cc} \frac{1}{\eta_{i}\phi_{i}} & 0\\ 0 & \frac{f_{i}(\theta_{i})}{\eta_{i}} \end{array}\right)\right).$$

Assuming that the study estimates are all independent, we can write

(B2) $$\sqrt{N}\left(\left(\begin{array}{c} {\hat{\beta }}_{1}\\ \vdots \\ {\hat{\beta }}_{k}\\ {\hat{\phi }}_{1}^{2}\\ \vdots \\ {\hat{\phi }}_{k}^{2} \end{array}\right)-\left(\begin{array}{c} \beta_{1}\\ \vdots \\ \beta_{k}\\ \phi_{1}^{2}\\ \vdots \\ \phi_{k}^{2} \end{array}\right)\right)\overset{\;d}{\underset{}{\to }}\;N_{2k}\left(\left(\begin{array}{c} 0_{k}\\ 0_{k} \end{array}\right),\left(\begin{array}{cc} {\text{Diag}}_{k}\{1/\eta_{i}\phi_{i}\} & 0_{kk}\\ 0_{kk} & {\text{Diag}}_{k}\{f_{i}(\theta_{i})/\eta_{i}\} \end{array}\right)\right).$$

Recalling the definition of *β*_*F*_:

$$\beta_{F}=g(\beta_{1},...\beta_{k},\phi_{1},\text{⋯},\phi_{k})=\frac{\sum_{i}^{k}\eta_{i}\phi_{i}\beta_{i}}{\sum_{i}^{k}\eta_{i}\phi_{i}},$$

we obtain the following derivatives:

$$\begin{array}{ccc} \frac{d}{d\beta_{i}}\beta_{F} & =\frac{\eta_{i}\phi_{i}}{\sum_{i=1}^{k}\eta_{i}\phi_{i}} & \\ \frac{d}{d\phi_{i}}\beta_{F} & =\frac{\left(\sum_{i=1}^{k}\eta_{i}\phi_{i}\right)\eta_{i}\beta_{i}-\left(\sum_{i=1}^{k}\eta_{i}\phi_{i}\beta_{i}\right)\eta_{i}}{{\left(\sum_{i=1}^{k}\eta_{i}\phi_{i}\right)}^{2}}=\frac{\eta_{i}(\beta_{i}-\beta_{F})}{\sum_{i=1}^{k}\eta_{i}\phi_{i}}. \end{array}$$

Then, as long as *f*_*i*_(***θ***_*i*_)<*∞* for *i*=1,…,*k*, we can apply the delta method to B2, obtaining

$$\sqrt{N}\left[\left(\frac{\sum_{i}^{k}\eta_{i}{\hat{\phi }}_{i}{\hat{\beta }}_{i}}{\sum_{i}^{k}\eta_{i}{\hat{\phi }}_{i}}\right)-\left(\frac{\sum_{i}^{k}\eta_{i}\phi_{i}\beta_{i}}{\sum_{i}^{k}\eta_{i}\phi_{i}}\right)\right]\overset{\;d\;}{\underset{}{\to }}N\left(0,\frac{1}{\sum_{i}^{k}\eta_{i}\phi_{i}}+\frac{\sum_{i}^{k}\eta_{i}{\left(\beta_{i}-\beta_{F}\right)}^{2}f_{i}(\theta_{i})}{{\left(\sum_{i}^{k}\eta_{i}\phi_{i}\right)}^{2}}\right),$$

equivalently

(B3) $$\sqrt{N}\left({\hat{\hat{\beta }}}_{F}-\beta_{F}\right)\overset{\;d\;}{\underset{}{\to }}N\left(0,\frac{1}{\left(\sum_{i}^{k}\eta_{i}\phi_{i}\right)}\left[1+\frac{\sum_{i}^{k}\eta_{i}{\left(\beta_{i}-\beta_{F}\right)}^{2}f_{i}(\theta_{i})}{\sum_{i}^{k}\eta_{i}\phi_{i}}\right]\right).$$

Here, we notice that for some special cases when *f*_*i*_(***θ***_*i*_)/*ϕ*_*i*_=*c*, a constant, for *i*=1,…,*k*, this expression can be factorized out and the inflation factor can be then expressed as (1+*c* *ζ* ^2^), a function of the heterogeneity parameter *ζ* ^2^ defined in Section 3.3. The specific form of *f*_*i*_(***θ***_*i*_) for some common effect estimators of continuous outcomes are provided in Appendix E.

## APPENDIX C ALTERNATIVE EXPRESSION FOR ζ ^2^ (SECTION 3.3)

### C.1

To simplify calculations, we write $w_{i}=\sigma_{i}^{-2}/\sum_{i=1}^{k}\sigma_{i}^{-2}$. Using this notation, we notice that $\sum_{i=1}^{k}w_{i}=1$ and $\sum_{i=1}^{k}w_{i}\beta_{i}=\beta_{F}$. The parameter *ζ* ^2^ can then be written as

$$\begin{array}{ccc} \zeta^{2} & =\sum_{i=1}^{k}w_{i}{\left(\beta_{i}-\beta_{F}\right)}^{2}=\sum_{i=1}^{k}w_{i}\beta_{i}^{2}-2\sum_{i=1}^{k}w_{i}\beta_{i}\beta_{F}+\beta_{F}^{2} & \\ & =\sum_{i=1}^{k}w_{i}\beta_{i}^{2}-\sum_{i=1}^{k}w_{i}\beta_{i}\beta_{F}\\ & =\sum_{i=1}^{k}w_{i}\left(\sum_{j=1}^{k}w_{j}\right)\beta_{i}^{2}-\sum_{i=1}^{k}w_{i}\beta_{i}\left(\sum_{j=1}^{k}w_{j}\beta_{j}\right)\\ & =\frac{1}{2}\sum_{i=1}^{k}\sum_{j=1}^{k}w_{i}w_{j}\beta_{i}^{2}+\frac{1}{2}\sum_{i=1}^{k}\sum_{j=1}^{k}w_{i}w_{j}\beta_{j}^{2}-\sum_{i=1}^{k}\sum_{j=1}^{k}w_{i}w_{j}\beta_{i}\beta_{j}\\ & =\frac{1}{2}\sum_{i=1}^{k}\sum_{j=1}^{k}w_{i}w_{j}{\left(\beta_{i}-\beta_{j}\right)}^{2}. \end{array}$$

## APPENDIX D MOMENT BASED ESTIMATOR OF ζ ^2^ (SECTION 4.1)

### D.1

Assuming known variances $\sigma_{1}^{2},\text{⋯},\sigma_{k}^{2}$, with $\sigma_{i}^{2}={\left(n_{i}\phi_{i}\right)}^{-1}$ for *i*=1,…,*k*, we start by calculating the expected value of a plug‐in estimate of *ζ* ^2^:

$$\begin{array}{ccc} E\left[\frac{\sum_{i=1}^{k}n_{i}\phi_{i}{\left({\hat{\beta }}_{i}-{\hat{\beta }}_{F}\right)}^{2}}{\sum_{i=1}^{k}n_{i}\phi_{i}}\right] & =E\left[\sum_{i=1}^{k}\frac{n_{i}\phi_{i}}{\Phi }{\hat{\beta }}_{i}^{2}-{\hat{\beta }}_{F}^{2}\right] & \\ & =\sum_{i=1}^{k}\frac{n_{i}\phi_{i}}{\Phi }\left(\mathrm{Var}[{\hat{\beta }}_{i}]+{\left(E[{\hat{\beta }}_{i}]\right)}^{2}\right)-\left(\mathrm{Var}[{\hat{\beta }}_{F}]+{\left(E[{\hat{\beta }}_{F}]\right)}^{2}\right)\\ & =\left(\sum_{i=1}^{k}\frac{n_{i}\phi_{i}}{\Phi }\beta_{i}^{2}-\beta_{F}^{2}\right)+\left(\frac{1}{\Phi }\sum_{i=1}^{k}n_{i}\phi_{i}\sigma_{i}^{2}-\frac{1}{\Phi }\right)\\ & =\sum_{i=1}^{k}\frac{n_{i}\phi_{i}}{\Phi }{\left(\beta_{i}-\beta_{F}\right)}^{2}+\frac{k-1}{\Phi }\\ & =\zeta^{2}+\frac{k-1}{\Phi }. \end{array}$$

Thus, an unbiased estimator of *ζ* ^2^ is then given by

$${\hat{\zeta }}^{2}=\frac{\sum_{i=1}^{k}\sigma_{i}^{-2}{\left({\hat{\beta }}_{i}-{\hat{\beta }}_{F}\right)}^{2}-(k-1)}{\sum_{i=1}^{k}\sigma_{i}^{-2}}=\frac{Q-(k-1)}{\Phi }.$$

## APPENDIX E ASYMPTOTIC VARIANCE OF ϕ FOR SOME COMMON EFFECT ESTIMATORS

### E.1

In this section, we derive the asymptotic variance of the information parameter *ϕ* for some common estimators of treatment effect for continuous outcomes. This asymptotic variance, denoted *f*(***θ***_*i*_), can then be plugged in estimators for the variance of ${\hat{\hat{\beta }}}_{F}$ described in Section 4.2 of the main paper.

#### E.1 Asymptotic variance of ϕ for the mean difference of independent groups

Following Borenstein,^48^ we first look at meta‐analyses of studies that compare the means of 2 independent groups, when an assumption of equal variances is made. Here, the effect size in the i*t* *h* study, *β*_*i*_=Δ_*i*_=*μ*_*i*,*X*_−*μ*_*i*,*Y*_, is estimated by ${\hat{\beta }}_{i}={\bar{X}}_{i}-{\bar{Y}}_{i}$, with $\mathrm{Var}({\hat{\beta }}_{i})=\sigma_{i}^{2}=(1/n_{i,X}+1/n_{i,Y})\varsigma_{i}^{2}$, where $\varsigma_{i}^{2}$ is the population variance, assumed to be the same for the 2 groups in study *i*, and *n*_*i*,*X*_ and *n*_*i*,*Y*_ are the respective sample sizes (with *n*_*i*_=*n*_*i*,*X*_+*n*_*i*,*Y*_). Here, we can write $\sigma_{i}^{2}={\left(n_{i}\phi_{i}\right)}^{-1}$ and $s_{i}^{2}={\left(n_{i}{\hat{\phi }}_{i}\right)}^{-1}$, with

(E1) $$\phi_{i}=g(\varsigma_{i}^{2})={\left[\left(\frac{1}{n_{i,X}/n_{i}}+\frac{1}{n_{i,Y}/n_{i}}\right)\varsigma_{i}^{2}\right]}^{-1}$$

and $\hat{\phi }=g({\hat{\varsigma }}_{i}^{2})$, where ${\hat{\varsigma }}_{i}^{2}$ is the pooled estimator of the variance, given by

$${\hat{\varsigma }}_{i}^{2}=\frac{\sum_{j=1}^{n_{i,X}}{\left(X_{ij}-{\bar{X}}_{i}\right)}^{2}+\sum_{j=1}^{n_{i,Y}}{\left(Y_{ij}-{\bar{Y}}_{i}\right)}^{2}}{n_{i}-2}=\frac{(n_{i,X}-1){\hat{\varsigma }}_{i,X}^{2}+(n_{i,Y}-1){\hat{\varsigma }}_{i,Y}^{2}}{n_{i,X}+n_{i,Y}-2},$$

and ${\hat{\varsigma }}_{i,X}^{2}$ and ${\hat{\varsigma }}_{i,Y}^{2}$ are the sample variances of the 2 groups in study *i*. To obtain an asymptotic distribution for ${\hat{\phi }}_{i}$, we start from a standard result for the sample variance:

$$\sqrt{n_{i}}({\hat{\varsigma }}_{i}^{2}-\varsigma_{i}^{2})\to_{d}N(0,(\kappa_{i}-1)\varsigma_{i}^{4}),$$

where *κ*_*i*_ denotes to the kurtosis in the population distribution (which we will also assume to be the same in the 2 groups for now). So then, keeping the sample size proportions (*n*_*i*,*X*_/*n*_*i*_ and *n*_*i*,*Y*_/*n*_*i*_) fixed, the derivative of (E1) is

$$\frac{d}{d\varsigma_{i}^{2}}g(\varsigma_{i}^{2})=-{\left(\frac{1}{n_{i,X}/n_{i}}+\frac{1}{n_{i,Y}/n_{i}}\right)}^{-1}{\left(\varsigma_{i}^{2}\right)}^{-2}.$$

The, applying the delta method, we conclude that

(E2) $$\sqrt{n_{i}}({\hat{\phi }}_{i}-\phi_{i})\to_{d}N\left(0,\frac{\kappa_{i}-1}{{\left(\frac{1}{n_{i,X}/n_{i}}+\frac{1}{n_{i,Y}/n_{i}}\right)}^{2}\varsigma_{i}^{4}}\right).$$

We notice that for studies with balanced design (*n*_*i*,*X*_=*n*_*i*,*Y*_), the information $\phi_{i}=1/4\varsigma_{i}^{2}$ and the asymptotic variance in the last expression reduces to $(\kappa_{i}-1)/4^{2}\varsigma_{i}^{4}$. If all studies in a meta‐analysis are balanced and the population variance and kurtosis can be assumed constant across all studies, then the inflation factor in (E2) is given by $\left(1+\frac{\kappa -1}{4\varsigma^{2}}\zeta^{2}\right)$. This is the expression used to estimate the inflation in type I error rate, produced when the estimation of standard errors is not taken into account, illustrated in Figure 2 of the main paper.

Now, when the assumption of equal variances is not made, the variance of ${\hat{\beta }}_{i}=\bar{X}-\bar{Y}$ is given by $\mathrm{Var}({\hat{\beta }}_{i})=\sigma_{i}^{2}=\varsigma_{i,X}^{2}/n_{iX}+\varsigma_{i,Y}^{2}/n_{i,Y}$, where $\varsigma_{i,X}^{2}$ and $\varsigma_{i,Y}^{2}$ are the population variances of the 2 groups in the i*t* *h* study.^48^ Similar to the case of equal variances, here, we can also write $\sigma_{i}^{2}={\left(n_{i}\phi_{i}\right)}^{-1}$ and $s_{i}^{2}={\left(n_{i}{\hat{\phi }}_{i}\right)}^{-1}$, now with

$$\phi_{i}=g(\varsigma_{i,X}^{2},\varsigma_{i,X}^{2})={\left(\frac{\varsigma_{i,X}^{2}}{n_{i,X}/n_{i}}+\frac{\varsigma_{i,Y}^{2}}{n_{i,Y}/n_{i}}\right)}^{-1}\;,$$

and ${\hat{\phi }}_{i}=g({\hat{\varsigma }}_{i,X}^{2},{\hat{\varsigma }}_{i,X}^{2})$. The asymptotic distribution for ${\hat{\varsigma }}_{i,X}^{2}$:

$$\sqrt{n_{i,X}}({\hat{\varsigma }}_{i,X}^{2}-\varsigma_{i,X}^{2})\to_{d}N(0,(\kappa_{i,X}-1)\varsigma_{i,X}^{4}),$$

can be expressed as

$$\sqrt{n_{i}}({\hat{\varsigma }}_{i,X}^{2}-\varsigma_{i,X}^{2})\to_{d}N\left(0,\frac{(\kappa_{i,X}-1)\varsigma_{i,X}^{4}}{n_{i,X}/n_{i}}\right),$$

and similarly for $\sqrt{n_{i}}({\hat{\varsigma }}_{i,Y}^{2}-\varsigma_{i,Y}^{2})$. Keeping the sample size proportions within each study (*n*_*i*,*X*_/*n*_*i*_ and *n*_*i*,*Y*_/*n*_*i*_) fixed, we can write

$$\sqrt{n_{i}}\left[\left(\begin{array}{c} {\hat{\varsigma }}_{i,X}^{2}\\ {\hat{\varsigma }}_{i,Y}^{2} \end{array}\right)-\left(\begin{array}{c} \varsigma_{i,X}^{2}\\ \varsigma_{i,Y}^{2} \end{array}\right)\right]\to_{d}N_{2}\left[0_{2},\left(\begin{array}{cc} \frac{(\kappa_{i,X}-1)\varsigma_{i,X}^{4}}{n_{i,X}/n_{i}} & 0\\ 0 & \frac{(\kappa_{i,Y}-1)\varsigma_{i,Y}^{4}}{n_{i,Y}/n_{i}} \end{array}\right)\right].$$

Taking derivatives, we find that

$$\nabla g(\varsigma_{i,X}^{2},\varsigma_{i,X}^{2})=-{\left(\frac{\varsigma_{i,X}^{2}}{n_{i,X}/n_{i}}+\frac{\varsigma_{i,Y}^{2}}{n_{i,Y}/n_{i}}\right)}^{-2}{\left(\frac{1}{n_{i,X}/n_{i}},\frac{1}{n_{i,Y}/n_{i}}\right)}^{T},$$

and applying the delta method, we obtain

$$\sqrt{n_{i}}({\hat{\phi }}_{i}-\phi_{i})\to_{d}N\left[0,\frac{\frac{(\kappa_{i,X}-1)\varsigma_{i,X}^{4}}{{\left(n_{i,X}/n_{i}\right)}^{3}}+\frac{(\kappa_{i,Y}-1)\varsigma_{i,Y}^{4}}{{\left(n_{i,Y}/n_{i}\right)}^{3}}}{{\left(\frac{\varsigma_{i,X}^{2}}{n_{i,X}/n_{i}}+\frac{\varsigma_{i,Y}^{2}}{n_{i,Y}/n_{i}}\right)}^{4}}\right].$$

#### E.2 LSSA for the mean difference between 2 matched samples

When the effect size in each study is the mean difference between 2 matched samples, we have that ${\hat{\beta }}_{i}={\bar{X}}_{i}-{\bar{Y}}_{i}$, with $\mathrm{Var}({\hat{\beta }}_{i})=\sigma_{i}^{2}=(\varsigma_{i,X}^{2}+\varsigma_{i,Y}^{2}-2\rho \varsigma_{i,X}\varsigma_{i,Y})/m_{i}$, where *ρ* denotes the population correlation between any 2 matched observations *X*_*i**j*_ and *Y*_*i**j*_ in study *i* and *m*_*i*_=*n*_*i*_/2 is the number of the paired observations.^48^ Assuming a bivariate normal distribution for the observations (*X*_*i**j*_,*Y*_*i**j*_), the following asymptotic distribution can be obtained for the sample variances ( ${\hat{\varsigma }}_{i,X}^{2},{\hat{\varsigma }}_{i,X}^{2}$) and sample covariance ( ${\hat{\varsigma }}_{i,XY}=\frac{1}{m_{i}}\sum_{j=1}^{m_{i}}(X_{ij}-{\bar{X}}_{i})(Y_{ij}-{\bar{Y}}_{i})$):

$$\sqrt{m_{i}}\left[\left(\begin{array}{c} {\hat{\varsigma }}_{i,X}^{2}\\ {\hat{\varsigma }}_{i,Y}^{2}\\ {\hat{\varsigma }}_{i,XY} \end{array}\right)-\left(\begin{array}{c} \varsigma_{i,X}^{2}\\ \varsigma_{i,Y}^{2}\\ \varsigma_{i,XY} \end{array}\right)\right]\to_{d}N_{3}\left[0_{3},\left(\begin{array}{ccc} 2\varsigma_{i,X}^{4} & 2\rho^{2}\varsigma_{i,X}^{2}\varsigma_{i,Y}^{2} & 2\rho \varsigma_{i,X}^{3}\varsigma_{i,Y}\\ 2\rho^{2}\varsigma_{i,X}^{2}\varsigma_{i,Y}^{2} & 2\varsigma_{i,Y}^{4} & 2\rho \varsigma_{i,X}\varsigma_{i,Y}^{3}\\ 2\rho \varsigma_{i,X}^{3}\varsigma_{i,Y} & 2\rho \varsigma_{i,X}\varsigma_{i,Y}^{3} & (1+\rho^{2})\varsigma_{i,X}^{2}\varsigma_{i,Y}^{2} \end{array}\right)\right].$$

Let $\sigma_{i}^{2}={\left(n_{i}\phi_{i}\right)}^{-1}=2(\varsigma_{i,X}^{2}+\varsigma_{i,Y}^{2}-2\varsigma_{i,XY})/n_{i}$ and

$$\phi_{i}=g(\varsigma_{i,X}^{2},\varsigma_{i,Y}^{2},\varsigma_{i,XY}^{2})={\left[2(\varsigma_{i,X}^{2}+\varsigma_{i,Y}^{2}-2\varsigma_{i,XY}^{2})\right]}^{-1},$$

with $\hat{\phi }=g({\hat{\varsigma }}_{i,X}^{2},{\hat{\varsigma }}_{i,Y}^{2},{\hat{\varsigma }}_{i,XY}^{2})$. Taking derivatives,

$$\nabla g(\varsigma_{i,X}^{2},\varsigma_{i,Y}^{2},\varsigma_{i,XY}^{2})=-\frac{1}{2}{\left(\varsigma_{i,X}^{2}+\varsigma_{i,Y}^{2}-2\varsigma_{i,XY}^{2}\right)}^{-2}{\left(1,1,-2\right)}^{T}$$

so we have, by the delta Method,

$$\sqrt{m_{i}}({\hat{\phi }}_{i}-\phi )\to_{d}N\left(0,\frac{2[\varsigma_{i,X}^{4}-8\rho \varsigma_{i,X}^{3}\varsigma_{i,Y}+2(1+2\rho^{2})\varsigma_{i,X}^{2}\varsigma_{i,Y}^{2}-8\rho \varsigma_{i,X}\varsigma_{i,Y}^{3}+\varsigma_{i,Y}^{4}]}{4{\left(\varsigma_{i,X}^{2}+\varsigma_{i,Y}^{2}-2\rho \varsigma_{i,X}\varsigma_{i,Y}\right)}^{4}}\right).$$
