# Supplementary material for: Addressing the estimation of standard errors in fixed effects meta‐analysis
Source: Stat Med. 2018 Mar 25;37(11):1788–809. doi: 10.1002/sim.7625 (PMC6001579; doi:10.1002/sim.7625)
Supplement: Supplementary file 1 — Coverage probability of 95% confidence intervals for β F using (a) a ‘naive’ estimator of the standard error, (b) the large sample size approximation (LSSA) estimator of the standard error and (c) the t‐statistic from the quasi‐F approach, along with the DerSimonian‐Laird estimator for the mean of random effects. The dotted horizontal line represents the asymptotic coverage for the ‘naive’ estimator, calculated analytically (see Appendix E.1). These results are from 10,000 simulations, with the gray bar re ecting the approximate Monte Carlo error. See detailed description of these estimators in Sections 4.1 and 4.2.1 of the main paper. Coverage probability of 95% confidence intervals for β F obtained from parametric bootstrap samples of size 1000: (a) a normal approximation using an empirical estimate of standard error, (b) the percentiles of the empirical distribution, (c) Bootstrap‐t based on a t‐statistic using a naive estimation of the standard error and (d) Bootstrap‐t based on a t‐statistic using the LSSA estimation of the standard error. These results are from 10,000 simulations, with the grey bar reflecting the expected Monte Carlo error. See detailed description of these estimators in Section 4.3 of the main paper. Coverage probability of 95% confidence intervals for ζ2 using: (a) an inverted probability interval from a non‐central χ2 distribution, (b) a normal approximation using an empirical estimate of the standard error from a parametric Bootstrap sample of size 1,000 and (c) the percentiles of the empirical distribution from the same parametric Bootstrap sample. These results are from 10,000 simulations, with the grey bar reflecting the expected Monte Carlo error. See detailed description of these estimators in Sections 4.1 to 4.3 of the main paper. [file SIM-37-1788-s001.pdf]

Supplemental material for "Addressing the estimation of standard errors in fixed effects meta-analysis"

Clara Dominguez-Islas*, Kenneth Rice†

1 Simulation study

We conducted a simulation study to evaluate and compare the different estimators of β_F and ζ^2 proposed in Section 4 of the paper. We present here a detailed description of the simulation settings, as well as the results obtained from different scenarios.

1.1 Settings

Our simulation settings consisted of meta-analysis with a small-to-medium number of studies ($k = 3, 5, 7, 15$) with fixed pre-specified effect-sizes ($\beta_1, ..., \beta_k$) that were evenly spread and centered around zero (so that $\beta_F = 0$) and with their absolute value determined by a pre-specified value of ζ^2 (0, 0.1, 0.4, 1, 2). All studies were set to have the same sample size ($n = 10, 20, 30, 40, 60, 80, 100, 500, 1000$) and the same population variance ($\varsigma_i^2 = 1$). A continuous Normal outcome was assumed, with the effect size given by the mean difference between two groups assuming equal variances and a balanced design. Under this assumptions, estimates of the effects were drawn from a Normal distribution

$$\hat{\beta}_i \sim N\left(\beta_i, \left(\frac{1}{n_i/2} + \frac{1}{n_i/2}\right)\varsigma_i^2\right) = N(\beta_i, 4/n_i), \text{ for } i = 1, ..., k;$$

while estimates of the variance where obtained from a scaled χ^2 distribution:

$$s_i^2 \sim \left(\frac{1}{n_i/2} + \frac{1}{n_i/2}\right)\left(\frac{\varsigma_i^2}{n_i - 2}\right)\chi^2_{n_i-2} = \frac{4}{n_i(n_i-2)}\chi^2_{n_i-2} \text{ for } i = 1, ..., k.$$

From each simulation we obtained the estimator $\hat{\hat{\beta}}_F$, as given in (19), along with 95% confidence intervals based on the following methods:

(a) A normal approximation using a "naive" estimator of $\widehat{SE}(\hat{\hat{\beta}}_F)$ (19) that does not account for the uncertainty in the estimation of the variances; also equivalent to assuming a common effect model.

(b) A normal approximation using the Large Sample Size Approximation (LSSA) for the variance of $\hat{\hat{\beta}}_F$ as given in (25), with the asymptotic variance of ϕ_i estimated by $(\kappa_i - 1)/4^2\hat{\varsigma}_i{}^4$ (C.13) and assuming a normal outcome ($\kappa_i = 3$, for $i = 1, ..., k$).

*Present address: Clara Domínguez Islas, Fred Hutchinson Cancer Research Center, 1100 Fairview Avenue N., M2-C200, PO Box 19024, Seattle, WA 98109-1024. Email: cdomingu@fredhutch.org

†Department of Biostatistics, University of Washington, Seattle WA, USA.

(c) A Student-t approximation based on the signed square root of the quasi-F statistic (30), with the degrees of freedom estimated by $\hat{\nu}$ (28).

(d) Parametric bootstrap estimation methods, as described in 1-4 from Section 4.2 of the paper, from 1000 bootstrap samples.

In addition to these, we also calculated the 95% confidence interval for the mean of the population of effects when using a random effects approach, based on the DerSimonian-Laird [1] estimator.

Estimates and 95% confidence intervals for ζ^2 were also computed from the simulated samples. Four different methods were implemented:

(a) A normal approximation using using a plugin estimate of the variance of $\hat{\zeta}^2$ as given in (17), with s_i^2 replacing σ_i^2.

(b) Inverting the probability interval of a non-central χ^2 distribution as described in (18), with s_i^2 replacing σ_i^2.

(c) A normal approximation using empirical estimate of the variance of $\hat{\zeta}^2$ from a parametric bootstrap of $\hat{\zeta}^2$.

(d) A percentile based interval from the empirical distribution of the parametric bootstrap sample of $\hat{\zeta}^2$.

1.2 Results

The coverage probability of the estimators described in D.1 was calculated from a total of 10,000 simulations for each combination of the simulation settings. Results on the coverage probability for 95% confidence intervals for β_F are presented in Figures 1.2 and 1.2 while results on 95% confidence intervals for ζ^2 are presented in Figure 1.2. The estimated Monte Carlo error is represented in each graph as a gray bar around the nominal 0.95 coverage probability.

References

[1] DerSimonian R, Laird N. Meta-analysis in clinical trials. *Control Clin Trials.* 1986;7(3):177 - 188.

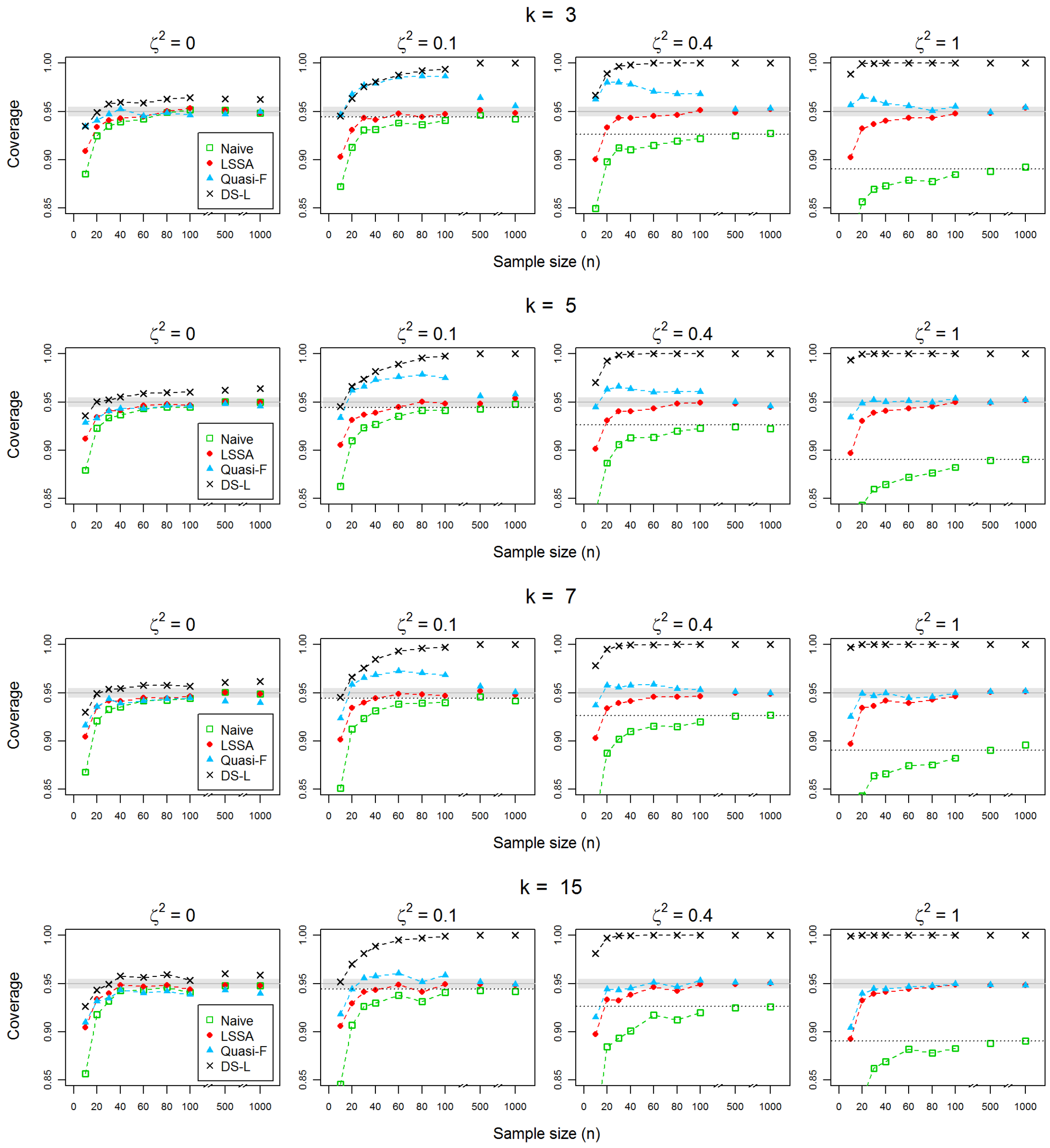

Figure 1.1: Coverage probability of 95% confidence intervals for β_F using (a) a 'naive' estimator of the standard error, (b) the large sample size approximation (LSSA) estimator of the standard error and (c) the t-statistic from the quasi-F approach, along with the DerSimonian-Laird estimator for the mean of random effects. The dotted horizontal line represents the asymptotic coverage for the 'naive' estimator, calculated analytically (see Appendix E.1). These results are from 10,000 simulations, with the gray bar reflecting the approximate Monte Carlo error. See detailed description of these estimators in Sections 4.1 and 4.2 of the main paper.

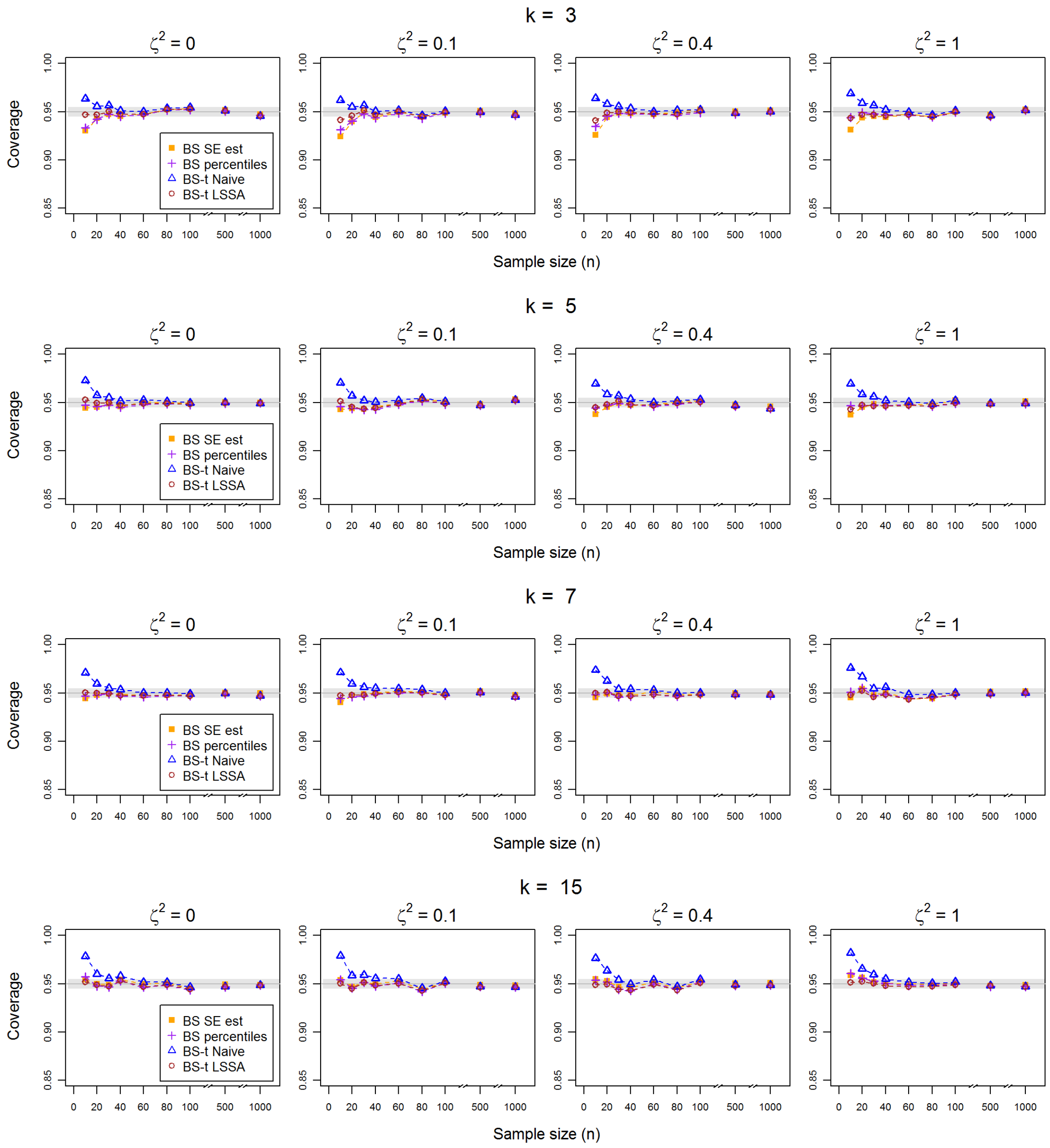

Figure 1.2: Coverage probability of 95% confidence intervals for β_F obtained from parametric bootstrap samples of size 1000: (a) a normal approximation using an empirical estimate of standard error, (b) the percentiles of the empirical distribution, (c) Bootstrap-t based on a t-statistic using a naive estimation of the standard error and (d) Bootstrap-t based on a t-statistic using the LSSA estimation of the standard error. These results are from 10,000 simulations, with the grey bar reflecting the expected Monte Carlo error. See detailed description of these estimators in Section 4.3 of the main paper.

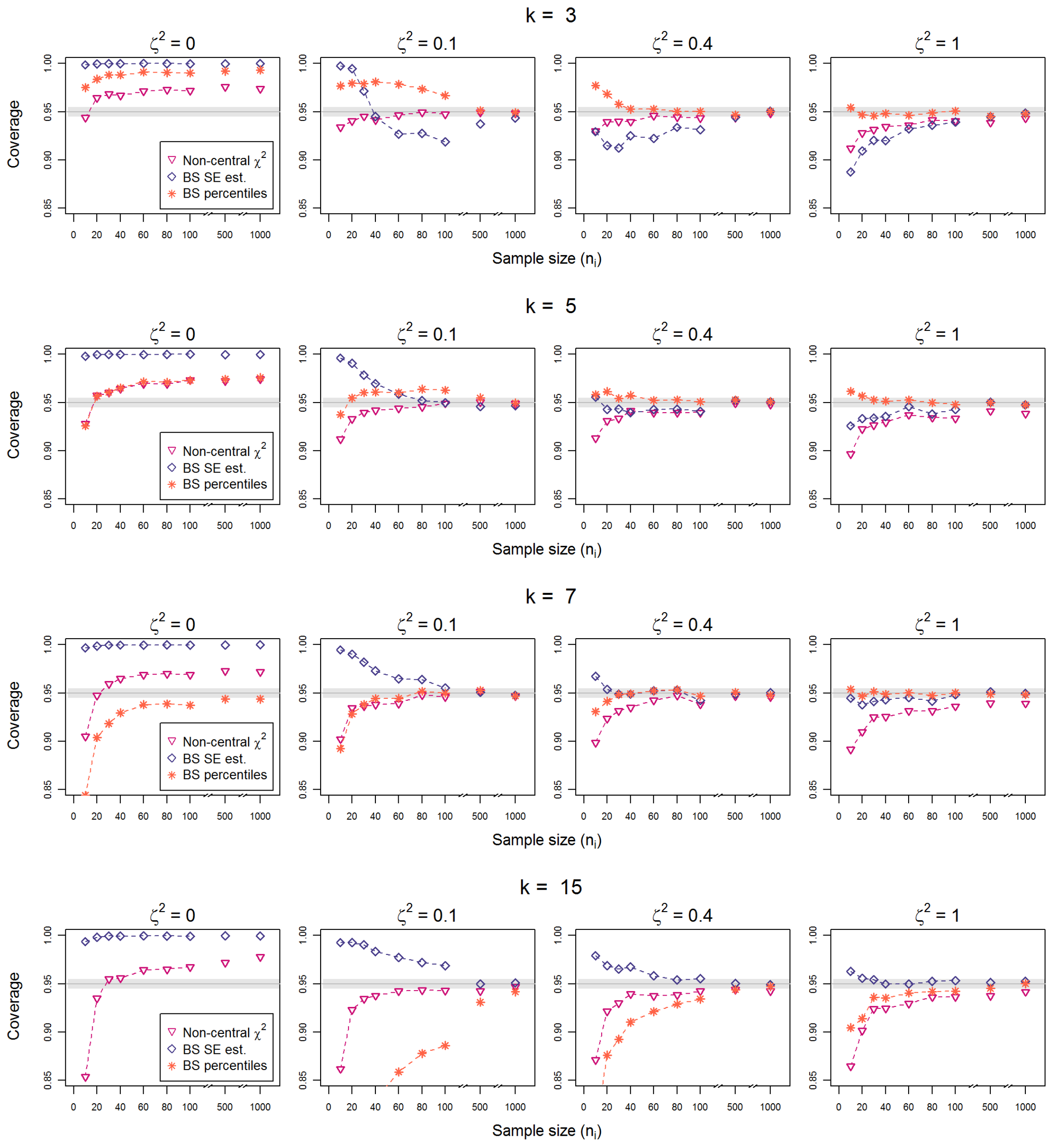

Figure 1.3: Coverage probability of 95% confidence intervals for ζ^2 using: (a) an inverted probability interval from a non-central χ^2 distribution, (b) a normal approximation using an empirical estimate of the standard error from a parametric Bootstrap sample of size 1,000 and (c) the percentiles of the empirical distribution from the same parametric Bootstrap sample. These results are from 10,000 simulations, with the grey bar reflecting the expected Monte Carlo error. See detailed description of these estimators in Sections 4.1 to 4.3 of the main paper.
